# Effect of accelerometer-measured physical activity on the association between atrial fibrillation and risk of dementia

**DOI:** 10.1016/j.tjpad.2026.100603

**Published:** 2026-05-19

**Authors:** Le Li, Mengtong Xu, Lingmin Wu, Zhicheng Hu, Limin Liu, Likun Zhou, Minghao Zhao, Yulong Xiong, Zhenhao Zhang, Lihui Zheng, Ligang Ding, Yan Yao

**Affiliations:** National Center for Cardiovascular Diseases, Chinese Academy of Medical Sciences, Peking Union Medical College, Fuwai hospital, Beijing, China

**Keywords:** Physical activity, Atrial fibrillation, Demantia, Risk factor

## Abstract

**Background:**

Atrial fibrillation (AF) independently increases dementia risk, but whether accelerometer-measured physical activity (PA) modifies this association remains unquantified, particularly against self-reported PA limitations.

**Methods:**

Prospective analysis of 91,795 UK Biobank participants with valid accelerometer data (median age 57, 42.9 % male; 2800 with baseline AF). We categorized whether measured activity met the standard recommendation [moderate-to-vigorous physical activity (MVPA) >_150 min/week]. Questionnaire-derived MVPA data from 353,643 UK Biobank participants (median age 57, male: 46.7 %) between 2006 and 2010 were used for validation. The primary outcome was the diagnosis of incident all-cause dementia. We also assessed correlation between accelerometer-derived and self-reported activity.

**Results:**

Over 7.6-year median follow-up, AF was significantly associated with a higher dementia risk [Adjusted hazard ratio (aHR): 1.76, 95 % confidential interval (CI): 1.51–2.05]. Guideline-adherent PA was associated with a lower AF-related dementia risk to non-significance (aHR: 1.36, 95 %CI: 0.96–1.91). Moreover, PA may be associated with higher protection effect on dementia risk in AF patients (aHR: 0.55, 95 % CI: 0.33–0.92) than in non-AF (aHR: 0.81, 95 % CI: 0.69–0.96), although without statistical difference (Pinteraction = 0.213). Correlation between accelerometer-derived and selfreported MVPA was weak (Spearman *r* = 0.155, 95 % CI: 0.148–0.162). Self-reported activity was not associated with a decreased risk of dementia in both AF and non-AF participants.

**Conclusion:**

Higher accelerometer-measured PA is associated with lower AF-associated dementia risk. Future prospective studies with extended follow-up and serial activity monitoring are needed to confirm these findings.

## 1 Introduction

Dementia is a leading global cause of impaired quality of life [1]. With increasing life expectancy and the accumulation of risk factors, the prevalence and incidence of dementia continue to rise steadily, presenting a major global health challenge [2]. Atrial fibrillation (AF), the most common sustained cardiac arrhythmia worldwide, is not only a significant risk factor for stroke and heart failure but has also been linked to cognitive impairment in numerous studies [[3], [4], [5]]. As the primary cause of stroke—a well-established major risk factor for dementia—AF contributes indirectly to dementia risk [6,7]. Furthermore, our recent pivotal study has confirmed a direct association between AF and cognitive decline, demonstrating that catheter ablation can mitigate this effect [8]. Importantly, AF and dementia also share several modifiable risk factors, such as advanced age, obesity, and diabetes [6,9].

Physical inactivity (PA) is recognized as one of 12 potentially modifiable risk factors collectively accounting for approximately 40 % of dementia cases in older adults [7]. Conversely, higher levels of PA are associated with a decreased risk of all-cause dementia (pooled relative risk [RR]: 0.80, 95 % confidence interval [CI] 0.77–0.84), Alzheimer’s disease (RR: 0.86, 95 % CI: 0.80–0.93), and vascular dementia (RR:0.79, 95 % CI: 0.66–0.95), even over extended follow-up periods [10]. However, the majority of evidence supporting this association relies on self-reported PA questionnaires, which are substantially limited by overestimation and show only modest correlations with objectively measured energy expenditure [11,12]. It is particularly important to further clarify the differences between self-reported physical activity and accelerometer-measured physical activity. Specifically, self-reported PA questionnaires mainly focus on assessing leisure-time activities, as participants are more likely to recall, and report structured or intentional exercise. In contrast, accelerometers are designed to capture all types of movement performed by the wearer, including not only leisure activities but also daily living activities, transport/mobility-related movement, and in particular, light intensity movements that are often overlooked or not recalled in self-report assessments.

Studies utilizing objective accelerometer-derived PA data to assess these relationships remain scarce, despite the superior accuracy offered by this methodology [13].

Optimizing PA management may be particularly crucial for individuals with AF. AF patients exhibit significantly worse physical function compared to those without AF, encompassing impairments in balance, gait speed, and coordination [14]. Moreover, their overall PA volume is markedly lower, likely attributable to deficits in peak oxygen consumption (VO_2_ max), ventilatory efficiency, and cardiac output [15,16]. Clarifying the specific benefits of PA in this population is therefore essential for developing evidence-based exercise recommendations for AF management. Previous research has established that PA can reduce complications like stroke and heart failure in AF patients [17]. Crucially, however, whether PA can mitigate or offset the increased dementia risk associated with AF remains unexplored.

In this study, we leveraged objective accelerometer data from nearly 100,000 participants in the prospective UK Biobank cohort study. We aimed to assess the associations between device-measured PA and incident dementia among individuals with and without AF. We hypothesized that higher levels of PA would attenuate the elevated risk of dementia conferred by AF.

## 2 Method

### 2.1 Data source

Our analytical sample was drawn from the prospective UK Biobank (UKB) cohort. At baseline recruitment (2006–2010), this sample comprised 502,129 community-dwelling adults. Briefly, UKB invited 9.2 million individuals aged 40–69 years residing within a 25-mile radius of 22 assessment centers across the UK; 5.4 % responded and participated in the baseline assessment [18,19]. At recruitment, questionnaires were completed and physical measurements were taken. Participants are followed for outcomes via linkage to national health datasets. All participants provided written informed consent, and the UKB study protocol received ethical approval from the North West Multi-centre Research Ethics Committee (11/NW/0382). Data access was granted under application number 394,593.

### 2.2 Accelerometer-derived physical activity

Between February 2013 and December 2015, 236,519 UK Biobank participants were invited to wear a wrist-worn accelerometer for seven consecutive days. Of these, 106,053 (44.8 %) agreed, and 103,695 (43.8 %) provided valid data [20]. PA volume was assessed using the Axivity AX3 wrist-worn triaxial accelerometer (Axivity Ltd., Newcastle upon Tyne, UK). The device recorded raw acceleration continuously at 100 Hz with a dynamic range of ±8 g, and signals underwent gravity calibration [20]. The overall mean acceleration (vector magnitude, derived from 5-second epoch aggregation of raw data) served as the measure of global PA volume [21]. Non-wear time was defined as periods ≥60 min where the standard deviation of the acceleration vector fell below 13.0 mg (milligravity) across all three axes [22].

As previously described [[23], [24], [25]], we quantified moderate-to-vigorous physical activity (MVPA) by summing all 5-second epochs where the mean acceleration was ≥ 100 mg. This threshold corresponds to activity of moderate intensity or higher for wrist-worn devices. Individual MVPA levels were then categorized based on established public health recommendations: Meeting the standard recommendation (≥ 150 min MVPA/week, ESC/AHA/WHO guidelines) [26–28].

### 2.3 Self-reported physical activity

Self-reported PA levels were also assessed using the short form of the International Physical Activity Questionnaire (IPAQ-SF) [29]. Consistent with previous methodology, we converted self-reported time spent walking, in moderate-intensity activities, and in vigorous-intensity activities into total weekly metabolic equivalent task-minutes (MET-min/week) [30]. We additionally categorized whether self-reported activity met ESC/AHA/WHO standard recommendations.

### 2.4 Study population

Two observational cohorts were established based on PA assessment modality: one utilizing objective evaluation via wrist-worn accelerometry and the other employing subjective evaluation through self-reported questionnaires. Both cohorts applied identical inclusion and exclusion criteria as detailed in **Supplementary Methods**. Comprehensive methodologies for handling missing data across study variables are detailed in the **Supplementary Methods** section. Follow-up commenced differentially by cohort: participants in the accelerometry cohort were tracked from accelerometer wear initiation, while the self-report cohort was followed from the date of UK Biobank enrollment consent. Person-years at risk were calculated for all participants from their cohort-specific baseline until the earliest occurrence of incident all-cause dementia (for dementia-specific analyses), all-cause death, or the end of follow-up on 31 December 2022.

### 2.5 Ascertainment of AF and dementia

AF cases were identified using the ​UK Biobank's​ first-occurrence diagnosis data (Field ID: 131,350). AF diagnosis was defined by the presence of the International Statistical Classification of Diseases and Related Health Problems, Tenth Revision (ICD-10) code I48. Cases were ascertained from linked health records spanning baseline assessment to follow-up, incorporating self-reported medical history, primary care data, hospital inpatient diagnoses, and death registry records.

Incident cases of all-cause dementia, Alzheimer’s disease (AD), and vascular dementia (VD) were determined using​UK Biobank's algorithmically defined outcome variables (Field IDs: 42,018, 42,020, 42,022, 42,024) [31]. Case identification relied on linked data from self-reported history (baseline), hospital admissions, and death registries. Outcomes were defined using relevant ICD-10 codes (detailed in **Table S1**). For each identified case, the earliest recorded diagnosis date across all data sources was designated as the dementia onset date.

### 2.6 Covariates

Demographic characteristics (age, sex, ethnicity, employment status) and lifestyle behaviors (smoking status, alcohol consumption) were ascertained through baseline self-report questionnaires. Comorbidities (hypertension, diabetes mellitus, cardiovascular diseases, and stroke) were identified via ICD-9/ICD-10 diagnostic codes derived from linked primary care and hospital admission records. Area-level socioeconomic deprivation was assessed using the Townsend Deprivation Index (TDI). We additionally incorporated two indices reflecting health and educational deprivation at the geographical level: the Health Score (quantifying premature mortality and health-related quality of life impairment) and the Education Score (measuring deprivation in educational attainment, skills development, and training access). The detailed definitions of covariates were shown in **Table S2**. Covariates for the statistical models were selected based on existing literature, biological plausibility, and availability of data as shown in the directed acyclic graph (**Figure S1**).

### 2.7 Statistical analysis

All analyses were conducted using R software (version 4.3.0). A two-sided p-value < 0.05 defined statistical significance. Continuous variable normality was assessed using the Kolmogorov-Smirnov test. Categorical variables are presented as frequencies (percentages). Normally distributed continuous variables are summarized as mean ± standard deviation (SD); non-normally distributed variables are presented as median (interquartile range, IQR). Group comparisons for continuous variables used the Mann‐Whitney U test; categorical variables used the chi-square test.

Our analysis proceeded in three sequential phases, aligning with the study design (**Figure S2**) [1]: Phase 1 (Association Validation):Evaluate the initial hypothesis that AF status (exposure) independently increases the risk of incident all-cause dementia (primary outcome) [2]. Phase 2 (Effect Modification): Determine if accelerometer-measured MVPA modifies the association identified in Phase 1 [3]. Phase 3 (Difference Analysis): Systematically evaluate whether PA reduces dementia risk differentially by AF status.

For time-to-event analysis, we used Kaplan-Meier estimation to construct cumulative incidence curves for dementia risk, stratified by AF status and MVPA categories. Between-strata differences were assessed using log-rank tests. In the present study, MVPA volume was categorized [1]: Guideline-Based: Below or at/above ESC/AHA/WHO standard (≥150 min/week MVPA) [2]. Distribution-Based: MVPA volume categorized into deciles due to its skewed distribution.

Cox proportional hazards regression models quantified associations between exposures and outcomes. The proportional hazards assumption was verified via Schoenfeld residual testing. We employed restricted cubic splines to model the potentially non-linear dose-response relationship between continuous MVPA volume and dementia risk. To account for the potential bias introduced by non-dementia death events, which may preclude the observation of dementia outcomes, we implemented Fine and Gray's subdistribution hazard models. In the present study, all-cause mortality was designated as the competing event. The population attributable fraction (PAF) was calculated to quantify the proportion of dementia risk attributable to modifiable physical activity levels in the population. PAF calculation methodology was described in prior publication [32]. Furthermore, subgroup analyses were performed for the associations between the adherence to MVPA standard guidelines and the risks of all-cause dementia: according to age, sex, employment status, and body mass index, hypertension, diabetes mellitus, CVD, stroke.

## 3 Results

### 3.1 Baseline characteristics

The accelerometer cohort comprised 91,795 participants following exclusions for poor-quality data, baseline dementia diagnosis, and other predefined criteria, including 2800 (3.1 %) with documented baseline AF (**Figure S3**). Participants had a median age of 57 years (IQR: 50–62) and 42.9 % were male. Baseline characteristics comparing AF versus non-AF participants appear in **Table S3**, while **Table S4** compares those achieving <150 versus ≥150 min/week MVPA. Participants were stratified into four groups by AF status and ESC/AHA/WHO standard guideline-based physical activity levels (AF-/PA-, AF-/PA+, AF+/PA-, AF+/PA+), with their characteristics detailed in Table 1. Dementia versus non-dementia group comparisons is shown in **Table S5**.

**Table 1:** Baseline characteristics.

Characteristics	Overall	AF-/PA-	AF-/PA+	AF+/PA-	AF+/PA+
**Total, n**	91,975	29,246	59,749	1177	1623
**Age, year**	57.0 (50.0, 62.0)	58.0 (51.0, 63.0)	62.0 (49.0, 62.0)	63.0 (59.0, 62.0)	62.0 (57.0, 66.0)
**Male, n (%)**	39,399 (42.9)	9221 (31.5)	28,299 (47.4)	662 (56.2)	1217 (75.0)
**Ethnicity, n (%)**					
British	83,483 (90.9)	26,689 (91.3)	54,171 (90.7)	1103 (93.7)	1520 (93.7)
Others	8312 (9.1)	2557 (8.7)	5578 (9.3)	74 (6.3)	103 (6.3)
**Employment status, n (%)**					
Employed	60,456 (65.9)	18,391 (62.9)	40,853 (68.4)	462 (39.3)	750 (46.2)
Retired or unemployed	31,339 (34.1)	10,855 (37.1)	18,896 (31.6)	715 (60.7)	873 (53.8)
**Education score**	8.2 (3.5, 16.6)	9.9 (4.3, 19.6)	7.6 (3.2, 15.2)	9.4 (3.9, 19.3)	7.0 (2.7, 14.6)
**Health score**	−0.3 (−0.8, 0.3)	−0.2 (−0.7, 0.3)	−0.3 (−0.8, 0.2)	−0.2 (−0.7, 0.4)	−0.3 (−0.8, 0.2)
**Townsend deprivation index**	−2.4 (−3.8, −0.2)	−2.5 (−3.8, −0.3)	−2.4 (−3.8, −0.1)	−2.5 (−3.8, −0.1)	−2.6 (−3.9, −0.5)
**BMI, kg/m^2^**	26.1 (23.6, 28.9)	27.0 (24.4, 30.5)	25.5 (23.3, 28.1)	28.1 (25.5, 32.3)	26.70 (24.4, 29.3)
**Smoking status, n (%)**					
Never	52,778 (57.5)	16,206 (55.4)	35,226 (59.0)	508 (43.2)	838 (51.6)
Former	32,659 (35.6)	10,417 (35.6)	20,945 (35.1)	591 (50.2)	706 (43.5)
Current	6358 (6.9)	2623 (9.0)	3578 (6.0)	78 (6.6)	79 (4.9)
**Drinking status, n (%)**					
Never	2700 (2.9)	1190 (4.1)	1426 (2.4)	43 (3.7)	41 (2.5)
Former	2492 (2.7)	964 (3.3)	1417 (2.4)	61 (5.2)	50 (3.1)
Current	86,603 (94.3)	27,092 (92.6)	56,906 (95.2)	1073 (91.2)	1532 (94.4)
**History of HTN, n (%)**	24,148 (26.3)	9161 (31.3)	13,457 (22.5)	741 (63.0)	789 (48.6)
**History of diabetes, n (%)**	3480 (3.8)	1669 (5.7)	1550 (2.6)	156 (13.3)	105 (6.5)
**History of CVD, n (%)**	6601 (7.2)	2318 (7.9)	3254 (5.5)	480 (40.8)	549 (33.8)
**History of stroke, n (%)**	1132 (1.2)	451 (1.5)	527 (0.9)	69 (5.9)	85 (5.2)

AF: atrial fibrillation; PA: physical activity; BMI: body mass index; HTN: hypertension; CVD: cardiovascular disease.

The self-reported cohort comprised 353,643 participants with median age 57 years (IQR: 49–63) and 46.7 % male. Participant characteristics stratified by MVPA level and dementia status appear in **Tables S6** and **Table S7**, respectively. Accelerometer-measured and self-reported MVPA distributions are compared in **Figure S4**. Guideline adherence differed substantially between cohorts: 66.7 % of accelerometer participants met ESC/AHA/WHO standards versus 55.8 % in the self-reported cohort.

### 3.2 Association of dementia in the accelerometer cohort

Of the 91,795 participants in the accelerometer cohort, 904 developed dementia over a median follow-up period of 7.6 years (IQR:7.0–8.1). The dementia incidence was significantly higher among AF participants than non-AF counterparts (2.75 % vs 0.93 %; log-rank *p* < 0.001; **Figure S5**). Multivariate-adjusted Cox regression demonstrated AF conferred a 41 % increased risk of dementia (HR 1.41, 95 % CI 1.10–1.80). This association remained consistent after accounting for competing mortality risk using Fine-Gray subdistribution hazards (sHR 1.36, 95 % CI 1.04–1.77) (**Table S8**).

**Fig. 1:**
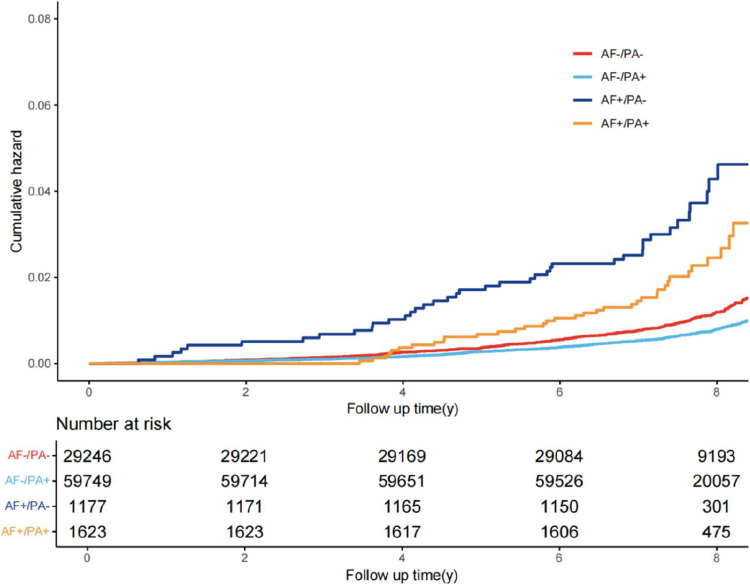
Cumulative risk of all-cause dementia among groups with different atrial fibrillation (AF) and physical activity (PA) status.

**Fig. 2:**
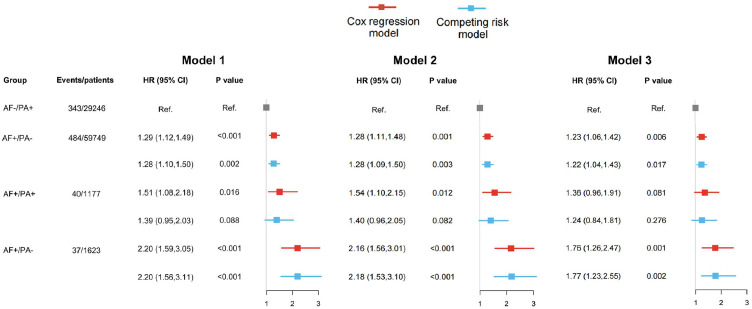
Association between atrial fibrillation (AF), physical activity (PA) and the risk of incident dementia, with AF-/PA+ as the reference group. Model 1: adjusted by: age, male, ethnicity; model 2:+ body mass index, health score, education score, Townsend deprivation index, employ status; model 3:+ smoke status, drink status,hypertension, diabetes, cardiovascular disease, and stroke.

Subsequent analyses demonstrated that accelerometer-measured PA significantly modified the relationship between AF and dementia risk in this cohort. Dementia incidence varied substantially across AF/PA status groups: the AF-/PA+ reference group exhibited the lowest rate (0.81 %), while AF+/PA- individuals showed the highest incidence (3.40 %), with a log-rank *P* < 0.001 (**Figures S6** and Fig. 1). The fully adjusted model, with AF-/PA+ as reference, revealed AF+/PA- conferred a 76 % increased dementia risk (HR: 1.76, 95 % CI: 1.26–2.47; *P* < 0.001). Crucially, AF+/PA+ exhibited attenuated risk that did not reach statistical significance (HR: 1.36, 95 % CI: 0.96–1.91; *P* = 0.081). This protective modification effect of PA persisted when accounting for competing mortality risks (Fig. 2).

Further analyses examining PA's impact on dementia risk across AF-defined subgroups revealed consistent patterns. When stratifying the total population, AF patients, and non-AF individuals by ESC/AHA/WHO guideline adherence status, those meeting activity recommendations exhibited significantly lower dementia incidence across all cohorts (log-rank *P* < 0.001 for all comparisons) (**Figure S7-S9**). Fully adjusted Cox models demonstrated protective associations throughout: guideline adherence corresponded to significant risk reductions in the overall population (HR: 0.79, 95 % CI: 0.68–0.92), AF subgroup (HR: 0.55, 95 % CI: 0.33–0.92), and non-AF subgroup (HR: 0.81, 95 % CI: 0.69–0.96). Although the magnitude of protection was numerically greater among AF patients, no statistically significant difference across subgroups was found (Pinteraction = 0.213). Critically, these protective relationships persisted through competing mortality risk analyses using Fine-Gray models (Table 2). Moreover, among AF patients, dementia incidence rates per 10,000 person-years were 45.4 (95 % CI 33.3–61.9) for those with <150 min/week MVPA versus 30.0 (95 % CI: 21.7–41.4) for guideline-adherent individuals. Non-AF patients exhibited lower overall incidence: 12.2 (95 % CI: 11.2–13.3) and 8.6 (95 % CI: 7.8–9.5) per 10,000 person-years in comparable activity categories. Using <150 min/week as reference, achieving ≥150 min/week MVPA conferred an absolute risk reduction of −15.4 cases per 10,000 person-years in AF patients (95 % CI: −30.5 to −0.3) compared to −3.6 cases in non-AF counterparts (95 % CI: −4.9 to −2.3), which may represent a greater absolute benefit in the AF cohort (**Table S9**).

**Table 2:** Associations between accelerometer-measured adherence of physical activity recommendations and all-cause dementia.

	Events/patients	Hazard ratio (95 % confidential interval)		
		Unadjusted model	Adjusted model	Compete model
**All participants**				
Below guideline	383/30,423	Ref.	Ref.	Ref.
At/above guideline	521/61,372	0.65 (0.56, 0.75)	0.79 (0.68, 0.92)	0.81 (0.69,0.94)
**With AF**				
Below guideline	40/1177	Ref.	Ref.	Ref.
At/above guideline	37/1623	0.54 (0.33, 0.89)	0.55 (0.33, 0.92)	0.58 (0.37,0.98)
**Without AF**				
Below guideline	343/29,246	Ref.	Ref.	Ref.
At/above guideline	484/59,749	0.68 (0.59, 0.79)	0.81 (0.69, 0.96)	0.83 (0.71, 0.98)

Adjusted model: including age, male, ethnicity, body mass index, health score, education score, Townsend deprivation index, employ status, smoke status, drink status, hypertension, diabetes, cardiovascular disease, and stroke. AF: atrial fibrillation.

In adjusted models assessing MVPA deciles as categorical exposures, dementia risk exhibited a nonlinear relationship across activity levels. For AF patients, risk progressively decreased through decile 7 (290–359 min/week), beyond which it plateaued with evidence of subtle attenuation at higher deciles. Among non-AF individuals, dementia risk similarly declined through decile 8 (360–454 min/week), stabilizing thereafter without material change (**Figure S10** and **Table S10-S12**). Sex-stratified analyses demonstrated consistently elevated dementia risk among males compared to females at comparable physical activity levels across all cohorts (overall, AF, and non-AF populations) (**Figure S11–13**). Moreover, the protective effect of meeting physical activity guidelines against dementia was significantly more pronounced in male participants (**Table S13**).

**Fig. 3:**
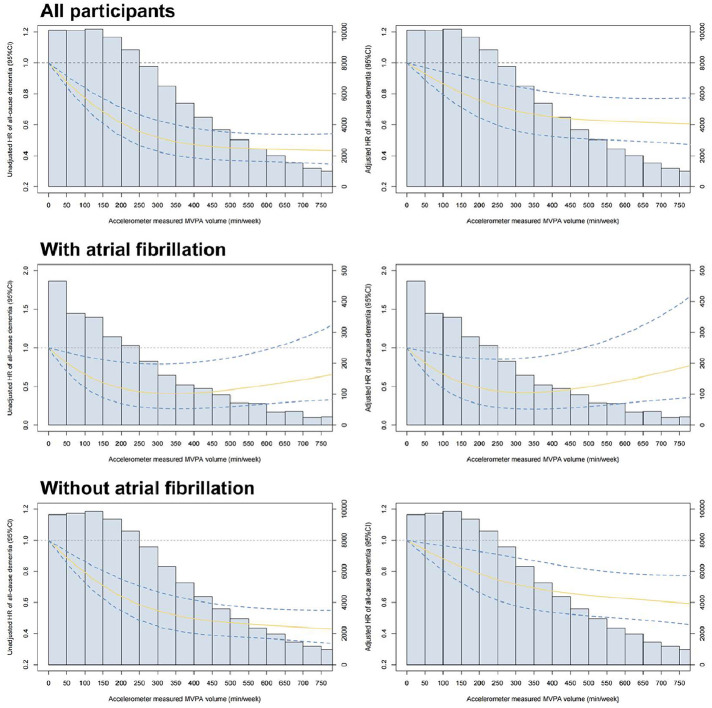
Dose-response relationship between accelerometer measured moderate to vigorous physical activity volume and risk of all-cause dementia in different population.

Dose-response analysis of accelerometer-measured MVPA as a continuous variable revealed distinct nonlinear patterns between cohorts (all P for non-linear < 0.05). Among AF patients, dementia risk decreased progressively with increasing MVPA volumes up to ∼300 min/week, beyond which the protective association plateaued and demonstrated diminishing returns at higher activity levels. For non-AF individuals, a linear reduction in dementia risk continued until ∼450 min/week of MVPA, after which the benefit curve similarly stabilized without further significant dementia risk reduction at extreme exercise volumes (Fig. 3).

### 3.3 Association of dementia in the self-reported cohort

Correlation analysis revealed a weak association between accelerometer-measured and self-reported MVPA volumes (Pearson's *r* = 0.155, 95 % CI: 0.148–0.162, *P* < 0.001; **Figure S14**). Contrary to objective measurements, analyses using self-reported data demonstrated no material difference in dementia incidence between guideline-adherent (≥150 min/week MVPA) and non-adherent groups, observed consistently across the overall population and AF/non-AF subgroups (**Figure S15-S17**). Notably, adjusted Cox models revealed paradoxical associations. Among AF patients, meeting activity guidelines showed a non-significant trend toward increased dementia risk versus non-adherent counterparts (HR: 1.09, 95 % CI: 0.85–1.39). More concerningly, non-AF participants meeting guidelines exhibited significantly elevated dementia risk (HR: 1.07, 95 % CI: 1.01–1.14) (**Table S14**).

Dose-response analyses revealed divergent relationships between MVPA volume and dementia risk by AF status. Among non-AF participants, accelerometer-measured MVPA demonstrated a paradoxical positive association with dementia risk, where increasing activity levels correlated with progressively elevated risk. For AF patients, no significant overall relationship emerged; however, a concerning risk elevation materialized at extreme activity volumes (>450 min/week MVPA) (**Figure S18**). These curvilinear patterns were reinforced when analyzing MVPA deciles as categorical exposures, confirming heightened vulnerability at supra-physiological activity thresholds in both subgroups (**Figure S19, Table S15–17**).

### 3.4 Subgroup analysis and effect modification

As shown in Table 3, using MVPA ≥ 150 min/week as the reference, the PAF for dementia associated with physical inactivity was 14.6 % (95 % CI 9.8–19.5 %) in the accelerometer cohort. The absolute PAF was greater among AF participants (16.3 %, 95 % CI 2.6–27.7 %) than non-AF individuals (13.6 %, 95 % CI 8.6–18.7 %), suggesting physical inactivity may contribute proportionally more to dementia risk in the AF population. Notably, no significant PAF was observed in the self-reported cohort.

**Table 3:** Population attributable fraction of physical activity for all-cause dementia risk stratified by atrial fibrillation status.

	Accelerometer measured cohort		Self-reported cohort	
	Event rate ratio	PAF (95 % CI)	Event rate ratio	PAF (95 % CI)
**All participants**				
Below guideline	1.48	14.6 (9.8, 19.5)	0.98	−3.1 (−5.6, −0.6)
At/above guideline	Ref.	Ref.	Ref.	Ref.
**With AF**				
Below guideline	1.49	16.3 (2.6, 27.7)	1.07	−2.8 (−10.6, 5.0)
At/above guideline	Ref.	Ref.	Ref.	Ref.
**Without AF**				
Below guideline	1.44	13.6 (8.6, 18.7)	0.97	−3.1 (−5.6, −0.5)
At/above guideline	Ref.	Ref.	Ref.	Ref.

PAF: Population attributable fraction; CI: confidential interval.

Subgroup analyses revealed a significant interaction between diabetes status and association of PA and dementia risk (Pinteraction = 0.039). Specifically, among non-AF participants with diabetes, guideline-adherent PA was associated with substantially reduced dementia risk (HR: 0.50, 95 % CI: 0.30–0.84) versus non-diabetics (HR: 0.87, 95 % CI: 0.74–1.01). No significant effect modifications were observed for age, sex, BMI, employment status, hypertension, cardiovascular disease, or stroke history (**Table S18**).

## 4 Discussion

Leveraging large-scale accelerometer-derived physical activity data from wrist-worn devices in nearly 100,000 participants, we demonstrate that AF significantly increases dementia risk, while increased physical activity may substantially attenuate this association. Our analysis revealed a robust protective relationship between higher PA levels and reduced all-cause dementia incidence, with this effect potentially more pronounced among AF patients compared to non-AF individuals. Crucially, analyses using self-reported PA data showed no significant association with incident dementia, highlighting the critical importance of objective measurement methodologies in physical activity research.

Our study substantiates and extends prior evidence demonstrating that accelerometer-derived PA is associated with reduced dementia risk. While previous investigations examining PA's neuroprotective effects have yielded conflicting conclusions, recent objective assessments provide compelling evidence. A dose-response analysis using accelerometer data revealed a significant inverse relationship between PA intensity and dementia risk [33]. Furthermore, Ning et al. demonstrated that objectively measured "weekend warrior" PA patterns may counteract dementia risk associated with prolonged sedentariness [34]. Contrastingly, the Whitehall II cohort study with 28-year follow-up of ∼10,000 participants found no neuroprotective association – potentially explained by reverse causation bias [35]. Their serial assessments detected declining PA levels during dementia's preclinical phase, suggesting reduced activity may be an early manifestation rather than causal factor. This discrepancy likely stems from methodological limitations: Whitehall II relied exclusively on self-reported PA, which is vulnerable to recall bias and misreporting [35]. Supporting this interpretation, Khurshid et al. documented minimal correlation between accelerometer-derived and self-reported MVPA [*r* = 0.16 (95 %CI:0.16–0.17)], with protective associations of AF observed only in accelerometer-verified cohorts [36]. It is important to stress that physical activity questionnaires and accelerometers are not measuring the same thing. While we have highlighted the poor correlation between self-report measures and accelerometer-derived PA, we further note that PA questionnaires may only assess a subset of an individual’s physical activity - for example, leisure-time activities - whereas accelerometers can capture all movement the wearer engages in, regardless of the context (e.g., occupational activity, household chores, or spontaneous movement in addition to leisure exercise) [37]. Our findings reinforce this critical methodological insight. We observed a similarly weak accelerometer-self-report correlation (*r* = 0.155) and diametrically opposed dementia risk associations: protective effects in accelerometer-measured PA versus paradoxical risk elevation in self-reported data. Collectively, these results underscore the necessity of objective PA measurement in dementia research to avoid measurement-error bias and spurious associations.

A key contribution of this study lies in systematically investigating the interrelationship between AF, dementia, and PA. While AF's dementia risk elevation is well-established [4], research on non-traditional interventions like PA for dementia prevention in AF patients remains limited beyond conventional rhythm control strategies [38]. More importantly, a relevant and so-far unexplored question is whether a PA can counteract the damaging influence of existing risk factors, such as AF, on incident dementia. In our study, we demonstrate that AF patients achieving guideline-recommended PA levels exhibited non-significant dementia risk elevation (HR 1.36, 95 % CI 0.96–1.91), indicating PA's critical role in mitigating AF-related dementia risk. Notably, in patients with AF who met the recommended physical activity levels, we observed no statistically significant difference in dementia risk. While this finding may be partly explained by the relatively limited number of incident dementia cases and restricted statistical power, our results still support the notion that sufficient physical activity may at least partially offset the excess dementia risk associated with AF. This interpretation is consistent with the overall trend toward reduced dementia risk observed in physically active patients with AF. Furthermore, point estimates suggested enhanced protective effects of PA against dementia in AF patients compared to non-AF individuals, though statistical significance was not reached. These findings hold particular clinical significance given the distinct protective role of PA in AF populations, underscoring the necessity for prioritized exercise prescription protocols and individualized activity management within AF care pathways [39]. It is important to emphasize, however, that the level of physical activity practiced by AF patients is influenced by multiple factors, including comorbidities, patient willingness, and physicians' factors, as highlighted by relevant literature. A systematic review and literature analysis by Leggio et al. comprehensively summarized the role of exercise training in AF, noting that various individual and clinical factors can modulate patients' engagement in physical activity [40]. Consistent with this, a recent European Heart Rhythm Association survey by Mills et al. further confirmed that lifestyle and risk factor modification, including physical activity, in AF patients is shaped by a combination of patient-related and clinician-related factors, which may in turn affect the implementation and effectiveness of PA interventions for dementia prevention [41]. The observed decreased dementia risk may be partially mediated through PA's established capacity to reduce stroke incidence—a key dementia pathway—particularly in AF patients where stroke risk is inherently elevated [36].

While the precise neurobiological mechanisms through which PA mitigates dementia risk remains incompletely characterized, multimodal neuroimaging provides compelling evidence of its structural and functional neuroprotective effects. Dove et al. demonstrated through serial MRI that PA counteracts accelerated brain aging in diabetic populations [42]. Erickson et al. established that aerobic exercise training induces a 2 % increase in anterior hippocampal volume, with concomitant improvements in spatial memory [43]. Complementing these findings, Hou et al. reported significant positive associations between physical activity levels and gray matter volume in 10 cerebral regions (Cohen's *d* = 0.004–0.108; *P* < 0.05), including hippocampal enlargement (β = 0.05, 95 % CI 0.02–0.08) [44]. Critically, Akinci et al. revealed through combined PET-MRI analyses that higher physical activity correlates with lower amyloid-β deposition—a cardinal dementia pathology[45]—in a dose-dependent manner, whereas sedentary behavior predicts reduced cortical thickness in Alzheimer's-vulnerable regions [46]. Furthermore, emerging evidence suggests physical frailty inversely correlates with integrity of cortical structures implicated in dementia pathogenesis, including bilateral bankssts, fusiform gyri, and inferior/middle temporal regions [47]. Future longitudinal studies integrating multimodal neuroimaging assessments—particularly in AF cohorts—will elucidate the underlying neurobiological mechanisms through which physical activity attenuates dementia risk.

Current pharmacological interventions for dementia prevention remain limited, particularly for high-risk populations like AF patients. While cardiovascular risk factor management offers partial protection, no targeted therapies exist to mitigate AF-specific neurocognitive decline. Alarmingly, over 40 % of dementia cases may be preventable through modifiable risk factors [7,48,49], yet scalable interventions remain elusive. Our study provides novel evidence that accelerometer-measured PA at guideline-recommended levels (≥150 min/week MVPA) significantly attenuates dementia risk, particularly in AF patients. The integration of wearable technology now enables precise activity monitoring—creating unprecedented opportunities for implementing personalized prevention strategies. Adherence to WHO-recommended activity levels proves feasible across middle-aged and older populations, with our accelerometer cohort showing 66.7 % compliance. Current guidelines already recognize physical activity as fundamental to cardiovascular disease prevention across primary, secondary, and tertiary stages [27,50]; our findings specifically support its formal integration into dementia prevention protocols for AF patients. Given the absence of pharmaceutical prevention options for dementia [7], PA represents a promising non-pharmacological strategy to modify neurocognitive trajectories—potentially altering dementia's natural course in this vulnerable population.

### 4.1 Limitations

Several limitations in our study should be acknowledged. First, the UK Biobank cohort is not fully representative of the general population due to healthy volunteer selection bias. This may lead to underestimation of absolute risk and potentially attenuate risk associations. Second, accelerometer data were obtained at a single timepoint, potentially overlooking longitudinal PA pattern changes. Third, despite comprehensive adjustment for confounders, residual confounding from unmeasured factors (e.g., subclinical cerebrovascular disease) cannot be excluded. Forth, the modest sample size of AF participants with accelerometer data (*n* = 2800) may limit statistical power for subgroup analyses. Fifth, the limited dementia cases observed in this study precluded subtype-specific analyses (e.g., vascular/Alzheimer's classification). Sixth, lack of distinction between paroxysmal and persistent AF in UK Biobank constrained our ability to assess differential associations between AF phenotypes and clinical outcomes. Seventh, while ICD codes can effectively identify moderate or severe dementia cases, milder cases are likely to be missed, which may lead to an underestimation of incident dementia events. Eighth, the relatively younger age of the cohort may further reduce the number of observed dementia events and limit the generalizability of our findings to the high-burden older population.

## 5 Conclusions

This large-scale longitudinal study utilizing objectively measured PA data demonstrates that AF significantly increases dementia risk, and this association is substantially attenuated by achieving guideline-recommended PA levels. Importantly, our findings suggest that AF patients may derive greater protective benefit against dementia from physical activity compared to non-AF individuals.

## Authorship / credit authorship contribution

L. Li, M. Xu and Y. Yao contributed to data acquisition, analysis, and interpretation, and drafted and critically reviewed the article for intellectual content. Y. Yao revised the article. L. Zheng, L. Ding, L. Wu, Z. Hu, L. Liu, L. Zhou, M. Zhao, Y. Xiong, Z. Zhang contributed to data analysis.

All authors contributed to the study and approved the final manuscript.

## Funding sources

This study was supported by National Major Science and Technology Project of National Health Commission (2024ZD0521900).

## Use of generative AI and AI-assisted technologies

The authors used generative AI (e.g., Deepseek) to proofread the English language. AI was not used to design figures, nor was it used to analyze data or interpret results. All final content and conclusions were reviewed and confirmed by the authors. The authors take full responsibility for the accuracy and integrity of the manuscript.

## Data statement

The data used in this study were obtained from the UK Biobank (https://www.ukbiobank.ac.uk/).

## CRediT authorship contribution statement

**Le Li:** Writing – original draft, Visualization, Data curation, Conceptualization. **Mengtong Xu:** Software, Methodology, Investigation, Formal analysis. **Lingmin Wu:** Visualization, Investigation, Data curation. **Zhicheng Hu:** Visualization, Validation, Resources. **Limin Liu:** Methodology, Investigation. **Likun Zhou:** Validation, Investigation, Data curation. **Minghao Zhao:** Investigation, Formal analysis. **Yulong Xiong:** Visualization, Supervision. **Zhenhao Zhang:** Visualization, Supervision. **Lihui Zheng:** Writing – review & editing, Visualization, Supervision. **Ligang Ding:** Writing – review & editing, Project administration. **Yan Yao:** Writing – review & editing, Methodology, Conceptualization.

## Declaration of competing interest

The authors declare that they have no known competing financial interests or personal relationships that could have appeared to influence the work reported in this paper.

## Appendix Supplementary materials

Image, application 1
