# Supplementary material for: Effect of accelerometer-measured physical activity on the association between atrial fibrillation and risk of dementia
Source: J Prev Alzheimers Dis. 2026 May 19;13(8):100603. doi: 10.1016/j.tjpad.2026.100603 (PMC13213870; doi:10.1016/j.tjpad.2026.100603)
Supplement: Supplementary file 1 [file mmc1.docx]

**Supplementary Material**

**Supplementary methods**

**Inclusion criteria:**

To preserve cohort integrity, all eligible participants identified during initial screening were comprehensively included in the primary analytic cohort.

**Exclusion criteria:**

1. **Exclude for poor data quality:**

- Participants undergoing wrist-worn accelerometer assessment must satisfy rigorous data quality criteria: 1) Minimum 72 hours of multiday recordings capturing full 24-hour cycles; 2) Accelerometer output magnitudes within manufacturer-specified operational ranges confirming device integrity; 3) Continuous data streams without interruption artifacts; 4) Sufficient raw signal resolution for calibration validation; 5) Stationary calibration periods demonstrating all three sensor axes within ±300 mg gravitational stability thresholds.
- In the self-reported cohort, participants responding to the physical activity assessment question—"How many minutes did you usually spend doing activities on a typical DAY?"—underwent rigorous data validation: 1) Responses <0 minutes or >1440 minutes/day were automatically excluded as physiologically implausible; 2) Values <10 or >300 minutes/day triggered confirmation prompts to verify accuracy; 3) Non-respondents and participants answering "Do not know" or "Prefer not to answer" were systematically excluded.

1. **Exclude for survival concerns:** 1) Loss to follow-up, which precludes endpoint ascertainment; 2) Baseline dementia diagnosis, which affects incident dementia assessment; 3) Accelerometer data collection preceding AF diagnosis, thus confounding temporal associations between physical activity and AF-related outcomes.
2. **Exclude for missing data:** Missing covariate data—predominantly involving demographic characteristics and behavioral factors (e.g., smoking status, alcohol consumption)—represented the primary source of data incompleteness in this study.

**Missing data handling methodologies**

Missing covariate data were handled through: categorical variable exclusion; continuous variable treatment via tiered approach including outlier mean imputation, feature exclusion (>30% missing), mean substitution (<5% missing), and multiple imputation (5-30% missing). Multiple imputation addresses missing data uncertainty by replacing missing values with multiple plausible estimates, generating m complete datasets. Unlike single imputation, MI incorporates estimation variability through Rubin's rules: (1) analyze each imputed dataset separately, (2) pool parameter estimates (e.g., regression coefficients), and (3) compute valid standard errors that account for within- and between-imputation variance. Imputation models predict missing values using all available variables meeting these criteria: (1) Predictive of missingness mechanism; (2) Associated with the incomplete variable; (3) Related to the analysis outcome(s). Multiple imputation analyses were conducted using the Multivariate Imputation by Chained Equations (MICE) package within the R statistical environment.

| **Table S1.** ICD-10 Codes Used to Ascertain All-Cause and Cause-Specific Dementia Cases | | | | | |
| --- | --- | --- | --- | --- | --- |
| **ICD 10 Code** | **Code description** | **Alzheimer’s disease** | **Vascular dementia** | **Frontotemporal dementia** | **All-cause dementia** |
| A81.0 | Sporadic Creutzfeldt-Jakob disease | 0 | 0 | 0 | 1 |
| F00 | Dementia in Alzheimer's disease | 1 | 0 | 0 | 1 |
| F00.0 | Dementia in Alzheimer's disease with early onset | 1 | 0 | 0 | 1 |
| F00.1 | Dementia in Alzheimer's disease with late onset | 1 | 0 | 0 | 1 |
| F00.2 | Dementia in Alzheimer's disease, atypical or mixed type | 1 | 0 | 0 | 1 |
| F00.9 | Dementia in Alzheimer's disease, unspecified | 1 | 0 | 0 | 1 |
| F01 | Vascular dementia | 0 | 1 | 0 | 1 |
| F01.0 | Vascular dementia of acute onset | 0 | 1 | 0 | 1 |
| F01.1 | Multi-infarct dementia | 0 | 1 | 0 | 1 |
| F01.2 | Subcortical vascular dementia | 0 | 1 | 0 | 1 |
| F01.3 | Mixed cortical and sub-cortical vascular dementia | 0 | 1 | 0 | 1 |
| F01.8 | Other vascular dementia | 0 | 1 | 0 | 1 |
| F01.9 | Vascular dementia, unspecified | 0 | 1 | 0 | 1 |
| F02 | Dementia in other diseases classified elsewhere | 0 | 0 | 0 | 1 |
| F02.0 | Dementia in Picks disease | 0 | 0 | 1 | 1 |
| F02.1 | Dementia in Creutzfeldt-Jacob disease | 0 | 0 | 0 | 1 |
| F02.2 | Dementia in Huntington’s disease | 0 | 0 | 0 | 1 |
| F02.3 | Dementia in Parkinson’s disease | 0 | 0 | 0 | 1 |
| F02.4 | Dementia in HIV disease | 0 | 0 | 0 | 1 |
| F02.8 | Dementia in other specified diseases classified elsewhere | 0 | 0 | 0 | 1 |
| F03 | Unspecified dementia | 0 | 0 | 0 | 1 |
| F05.1 | Delirium superimposed on dementia | 0 | 0 | 0 | 1 |
| F10.6 | Mental and behavioral disorders due to use of alcohol - amnesic syndrome | 0 | 0 | 0 | 1 |
| G30 | Alzheimer’s disease | 1 | 0 | 0 | 1 |
| G30.0 | Alzheimer’s disease with early onset | 1 | 0 | 0 | 1 |
| G30.1 | Alzheimer’s disease with late onset | 1 | 0 | 0 | 1 |
| G30.8 | Other Alzheimer's disease | 1 | 0 | 0 | 1 |
| G30.9 | Alzheimer's disease unspecified | 1 | 0 | 0 | 1 |
| G31.0 | Circumscribed brain atrophy | 0 | 0 | 1 | 1 |
| G31.1 | Senile degeneration of brain | 0 | 0 | 0 | 1 |
| G31.8 | Other specified degenerative diseases of nervous system | 0 | 0 | 0 | 1 |
| I67.3 | Binswanger's disease | 0 | 1 | 0 | 1 |

| **Table S2.** Definitions of diseases in our study based on ICD-10 | |
| --- | --- |
| **Diseases** | **ICD-10 code** |
| Atrial fibrillation | I48 |
| Hypertension | I10 |
| Diabetes | E10-E14 |
| Cardiovascular diseases | I01-02, I05-I09, I20-I25, I30-31, I33-I38, I40-I47, I49, I50 |
| Stroke | I60-I64 |

**Table S3.** Baseline characteristics between AF and non-AF individuals

| **Characteristics** | **Total** | **Non-AF** | **AF** | **P value** |
| --- | --- | --- | --- | --- |
| **Total, n** | 91975 | 88995 | 2800 |  |
| **Age, year** | 57.0 (50.0, 62.0) | 57.0 (50.0, 62.0) | 62.5 (58.0, 66.0) | < 0.001 |
| **Male, n (%)** | 39399 (42.9) | 37520 (42.2) | 1879 (67.1) | < 0.001 |
| **Ethnicity, n (%)** |  |  |  | < 0.001 |
| British‌ | 83483 (90.9) | 80860 (90.9) | 2623 (93.7) |  |
| Others | 8312 (9.1) | 8135 (9.1) | 177 (6.3) |  |
| **Employed status, n (%)** |  |  |  | < 0.001 |
| Employed | 60456 (65.9) | 59244 (66.6) | 1212 (43.3) |  |
| Retired or unemployed | 31339 (34.1) | 29751 (33.4) | 1588 (56.7) |  |
| **Education score** | 8.2 (3.5, 16.6) | 8.2 (3.5, 16.6) | 7.9 (3.1, 16.6) | 0.023 |
| **Health score** | -0.3 (-0.8, 0.3) | -0.3 (-0.8, 0.3) | -0.3 (-0.8, 0.3) | 0.778 |
| **Townsend deprivation index** | -2.4 (-3.8, -0.2) | -2.4 (-3.8, -0.2) | -2.5 (-3.9, -0.4) | 0.078 |
| **BMI, kg/m^2^** | 26.1 (23.6, 28.9) | 26.0 (23.6, 28.9) | 27.0 (24.9, 30.4) | < 0.001 |
| **Smoking status, n (%)** |  |  |  | < 0.001 |
| Never | 52778 (57.5) | 51432 (57.8) | 1346 (48.1) |  |
| Former | 32659 (35.6) | 31362 (35.2) | 1297 (46.3) |  |
| Current | 6358 (6.9) | 6201 (7.0) | 157 (5.6) |  |
| **Drinking status, n (%)** |  |  |  | < 0.001 |
| Never | 2700 (2.9) | 2616 (2.9) | 84 (3.0) |  |
| Former | 2492 (2.7) | 2381 (2.7) | 111 (4.0) |  |
| Current | 86603 (94.3) | 83998 (94.4) | 2605 (93.0) |  |
| **History of HTN, n (%)** | 24148 (26.3) | 22618 (25.4) | 1530 (54.6) | < 0.001 |
| **History of diabetes, n (%)** | 3480 (3.8) | 3219 (3.6) | 261 (9.3) | < 0.001 |
| **History of CVD, n (%)** | 6601 (7.2) | 5572 (6.3) | 1029 (36.8) | < 0.001 |
| **History of stroke, n (%)** | 1132 (1.2) | 978 (1.1) | 154 (5.5) | < 0.001 |

AF: atrial fibrillation; BMI: body mass index; HTN: hypertension; CVD: cardiovascular disease.

| **Table S4.** Baseline characteristics between different physical activity status | | | | |
| --- | --- | --- | --- | --- |
| **Characteristics** | **Total** | **MVPA ≥ 150 min/week** | **MVPA < 150 min/week** | **P value** |
| **Total, n** | 91975 | 61372 | 30423 |  |
| **Age, year** | 57.0 (50.0, 62.0) | 56.0 (49.0, 62.0) | 58.0 (51.0, 63.0) | < 0.001 |
| **Male, n (%)** | 39399 (42.9) | 29516 (48.1) | 9883 (32.5) | < 0.001 |
| **Ethnicity, n (%)** |  |  |  | < 0.001 |
| British‌ | 83483 (90.9) | 55691 (90.7) | 27792 (91.4) |  |
| Others | 8312 (9.1) | 5681 (9.3) | 2631 (8.6) |  |
| **Employed status, n (%)** |  |  |  | < 0.001 |
| Employed | 60456 (65.9) | 41603 (67.8) | 18853 (62.0) |  |
| Retired or unemployed | 31339 (34.1) | 19769 (32.2) | 11570 (38.0) |  |
| **Education score** | 8.2 (3.5, 16.6) | 7.62 (3.2, 15.2) | 9.90 (4.3, 19.6) | < 0.001 |
| **Health score** | -0.3 (-0.8, 0.3) | -0.3 (-0.8, 0.2) | -0.2 (-0.7, 0.3) | < 0.001 |
| **Townsend deprivation index** | -2.4 (-3.8, -0.2) | -2.4 (-3.8, -0.1) | -2.5 (-3.8, -0.3) | 0.001 |
| **BMI, kg/m^2^** | 26.1 (23.6, 28.9) | 25.6 (23.3, 28.1) | 27.1 (24.4, 30.6) | < 0.001 |
| **Smoking status, n (%)** |  |  |  | < 0.001 |
| Never | 52778 (57.5) | 36064 (58.8) | 16714 (54.9) |  |
| Former | 32659 (35.6) | 21651 (35.3) | 11008 (36.2) |  |
| Current | 6358 (6.9) | 3657 (6.0) | 2701 (8.9) |  |
| **Drinking status, n (%)** |  |  |  | < 0.001 |
| Never | 2700 (2.9) | 1467 (2.4) | 1233 (4.1) |  |
| Former | 2492 (2.7) | 1467 (2.4) | 1025 (3.4) |  |
| Current | 86603 (94.3) | 58438 (95.2) | 28165 (92.6) |  |
| **History of HTN, n (%)** | 24148 (26.3) | 14246 (23.2) | 9902 (32.5) | < 0.001 |
| **History of diabetes, n (%)** | 3480 (3.8) | 1655 (2.7) | 1825 (6.0) | < 0.001 |
| **History of CVD, n (%)** | 6601 (7.2) | 3803 (6.2) | 2798 (9.2) | < 0.001 |
| **History of stroke, n (%)** | 1132 (1.2) | 612 (1.0) | 520 (1.7) | < 0.001 |

MVPA: moderate-to-vigorous physical activity；BMI: body mass index; HTN: hypertension; CVD: cardiovascular disease

| **Table S5.** Baseline characteristics between dementia and non-dementia individuals | | | | |
| --- | --- | --- | --- | --- |
| **Characteristics** | **Total** | **Non-dementia** | **Dementia** | **P value** |
| **Total, n** | 91975 | 90891 | 904 |  |
| **Age, year** | 57.0 (50.0, 62.0) | 57.0 (50.0, 62.0) | 65.0 (61.0, 67.0) | < 0.001 |
| **Male, n (%)** | 39399 (42.9) | 38916 (42.8) | 483 (53.4) | < 0.001 |
| **Ethnicity, n (%)** |  |  |  | 0.167 |
| British‌ | 83483 (90.9) | 82649 (90.9) | 834 (92.3) |  |
| Others | 8312 (9.1) | 8242 (9.1) | 70 (7.7) |  |
| **Employed status, n (%)** |  |  |  | < 0.001 |
| Employed | 60456 (65.9) | 60185 (66.2) | 271 (30.0) |  |
| Retired or unemployed | 31339 (34.1) | 30706 (33.8) | 633 (70.0) |  |
| **Education score** | 8.2 (3.5, 16.6) | 8.2 (3.5, 16.6) | 8.7 (3.6, 17.1) | 0.276 |
| **Health score** | -0.3 (-0.8, 0.3) | -0.3 (-0.8, 0.3) | -0.2 (-0.7, 0.3) | 0.177 |
| **Townsend deprivation index** | -2.4 (-3.8, -0.2) | -2.4 (-3.8, -0.2) | -2.5 (-3.9, 0.1) | 0.975 |
| **BMI, kg/m^2^** | 26.1 (23.6, 28.9) | 26.1 (23.6, 28.9) | 26.3 (24.0, 29.1) | 0.029 |
| **Smoking status, n (%)** |  |  |  | < 0.001 |
| Never | 52778 (57.5) | 52343 (57.6) | 435 (48.1) |  |
| Former | 32659 (35.6) | 32244 (35.5) | 415 (45.9) |  |
| Current | 6358 (6.9) | 6304 (6.9) | 54 (6.0) |  |
| **Drinking status, n (%)** |  |  |  | 0.002 |
| Never | 2700 (2.9) | 2669 (2.9) | 31 (3.4) |  |
| Former | 2492 (2.7) | 2451 (2.7) | 41 (4.5) |  |
| Current | 86603 (94.3) | 85771 (94.4) | 832 (92.0) |  |
| **History of HTN, n (%)** | 24148 (26.3) | 23768 (26.2) | 380 (42.0) | < 0.001 |
| **History of diabetes, n (%)** | 3480 (3.8) | 3390 (3.7) | 90 (10.0) | < 0.001 |
| **History of CVD, n (%)** | 6601 (7.2) | 6450 (7.1) | 151 (16.7) | < 0.001 |
| **History of stroke, n (%)** | 1132 (1.2) | 1098 (1.2) | 34 (3.8) | < 0.001 |

BMI: body mass index; HTN: hypertension; CVD: cardiovascular disease.

| **Table S6.** Baseline characteristics between different physical activity status in the self-reported cohort | | | | |
| --- | --- | --- | --- | --- |
| **Characteristics** | **Total** | **MVPA ≥ 150 min/week** | **MVPA < 150 min/week** | **P value** |
| **Total, n** | 353643 | 197302 | 156341 |  |
| **Age, year** | 57.0 (49.0, 63.0) | 57.0 (49.0, 63.0) | 56.0 (49.0, 62.0) | < 0.001 |
| **Male, n (%)** | 165023 (46.7) | 92805 (47.0) | 72218 (46.2) | < 0.001 |
| **Ethnicity, n (%)** |  |  |  | < 0.001 |
| British‌ | 314396 (88.9) | 175937 (89.2) | 138459 (88.6) |  |
| Others | 39247 (11.1) | 21365 (10.8) | 17882 (11.4) |  |
| **Employed status, n (%)** |  |  |  | < 0.001 |
| Employed | 230801 (65.3) | 121677 (61.7) | 109124 (69.8) |  |
| Retired or unemployed | 122842 (34.7) | 75625 (38.3) | 47217 (30.2) |  |
| **Education score** | 11.1 (4.3, 20.6) | 11.0 (4.2, 20.3) | 11.3 (4.4, 21.0) | < 0.001 |
| **Health score** | -0.1 (-0.7, 0.4) | -0.1 (-0.7, 0.4) | -0.1 (-0.7, 0.4) | < 0.001 |
| **Townsend deprivation index** | -2.2 (-3.7, 0.3) | -2.3 (-3.7, 0.2) | -2.2 (-3.7, -0.4) | < 0.001 |
| **BMI, kg/m^2^** | 26.6 (24.1, 29.5) | 26.3 (23.8, 29.0) | 26.9 (24.4, 30.2) | < 0.001 |
| **Smoking status, n (%)** |  |  |  | < 0.001 |
| Never | 196708 (55.6) | 109494 (55.5) | 87214 (55.8) |  |
| Former | 121607 (34.4) | 69370 (35.2) | 52237 (33.4) |  |
| Current | 35328 (10.0) | 18438 (9.4) | 16890 (10.8) |  |
| **Drinking status, n (%)** |  |  |  | < 0.001 |
| Never | 13500 (3.8) | 7023 (3.6) | 6477 (4.1) |  |
| Former | 11699 (3.3) | 6216 (3.2) | 5483 (3.5) |  |
| Current | 328444 (92.9) | 184063 (93.3) | 144381 (92.4) |  |
| **History of HTN, n (%)** | 87441 (24.7) | 46300 (23.5) | 41141 (26.3) | < 0.001 |
| **History of diabetes, n (%)** | 10031 (2.8) | 4674 (2.4) | 5357 (3.4) | < 0.001 |
| **History of CVD, n (%)** | 16762 (4.7) | 8931 (4.5) | 7831 (5.0) | < 0.001 |
| **History of stroke, n (%)** | 4596 (1.3) | 2285 (1.2) | 2311 (1.5) | < 0.001 |

MVPA: moderate-to-vigorous physical activity；BMI: body mass index; HTN: hypertension; CVD: cardiovascular disease

| **Table S7.** Baseline characteristics between dementia and non-dementia individuals in the self-reported cohort | | | | |
| --- | --- | --- | --- | --- |
| **Characteristics** | **Total** | **Non-dementia** | **Dementia** | **P value** |
| **Total, n** | 353643 | 347854 | 5789 |  |
| **Age, year** | 57.0 (49.0, 63.0) | 57.0 (49.0, 62.0) | 65.0 (62.0, 67.0) | < 0.001 |
| **Male, n (%)** | 165023 (46.7) | 161880 (46.5) | 3143 (54.3) | < 0.001 |
| **Ethnicity, n (%)** |  |  |  | < 0.001 |
| British‌ | 314396 (88.9) | 309131 (88.9) | 5265 (91.0) |  |
| Others | 39247 (11.1) | 38723 (11.1) | 524 (9.1) |  |
| **Employed status, n (%)** |  |  |  | < 0.001 |
| Employed | 230801 (65.3) | 229289 (65.9) | 1512 (26.1) |  |
| Retired or unemployed | 122842 (34.7) | 118565 (34.1) | 4277 (73.9) |  |
| **Education score** | 11.1 (4.3, 20.6) | 11.1 (4.3, 20.6) | 13.0 (5.1, 23.5) | < 0.001 |
| **Health score** | -0.1 (-0.7, 0.4) | -0.1 (-0.7, 0.4) | -0.0 (-0.6, 0.5) | < 0.001 |
| **Townsend deprivation index** | -2.2 (-3.7, 0.3) | -2.3 (-3.7, 0.3) | -2.1 (-3.6, 0.7) | < 0.001 |
| **BMI, kg/m^2^** | 26.6 (24.1, 29.5) | 26.6 (24.1, 29.5) | 26.7 (24.4, 29.5) | < 0.001 |
| **Smoking status, n (%)** |  |  |  | < 0.001 |
| Never | 196708 (55.6) | 193999 (55.8) | 2709 (46.8) |  |
| Former | 121607 (34.4) | 119108 (34.2) | 2499 (43.2) |  |
| Current | 35328 (10.0) | 34747 (10.0) | 581 (10.0) |  |
| **Drinking status, n (%)** |  |  |  | < 0.001 |
| Never | 13500 (3.8) | 13160 (3.8) | 340 (5.8) |  |
| Former | 11699 (3.3) | 11336 (3.3) | 363 (6.3) |  |
| Current | 328444 (92.9) | 323358 (93.0) | 5086 (87.9) |  |
| **History of HTN, n (%)** | 87441 (24.7) | 85086 (24.5) | 2355 (40.7) | < 0.001 |
| **History of diabetes, n (%)** | 10031 (2.8) | 9637 (2.8) | 394 (6.8) | < 0.001 |
| **History of CVD, n (%)** | 16762 (4.7) | 16117 (4.6) | 645 (11.1) | < 0.001 |
| **History of stroke, n (%)** | 4596 (1.3) | 4364 (1.3) | 232 (4.0) | < 0.001 |

BMI: body mass index; HTN: hypertension; CVD: cardiovascular disease.

| **Table S8.** Cox regression of atrial fibrillation and dementia risk | | | | |
| --- | --- | --- | --- | --- |
|  | **Event rate** | **HR (95% CI)** | | |
|  |  | **Unadjusted model** | **Adjusted model** | **Compete model** |
| AF | 2.75% | 3.13 (2.48, 3.96) | 1.41 (1.10, 1.80) | 1.36 (1.04, 1.77) |
| Non-AF | 0.93% | Ref. | Ref. | Ref. |

The models were adjusted by age, male, ethnicity, body mass index, health score, education score, Townsend deprivation index, employ status, smoke status, drink status, hypertension, diabetes, cardiovascular disease, and stroke (fully model). AF: atrial fibrillation; HR: hazard ratio; CI: confidential interval.

| **Table S9.** Incidence event rate, incidence rate difference, and incidence rate ratio of dementia by atrial fibrillation status. | | | | | | |
| --- | --- | --- | --- | --- | --- | --- |
|  | **With AF** | | | **Without AF** | | |
|  | **Incidence event rate*** | **Incidence rate difference** | **Incidence rate ratio** | **Incidence event rate** | **Incidence rate difference** | **Incidence rate ratio** |
| **Below ESC/AHA/WHO recommendation** | 45.4 (33.3, 61.9) | Ref. | Ref. | 15.3 (13.8, 17.1) | Ref. | Ref. |
| **At/above ESC/AHA/WHO recommendation** | 30.0 (21.7, 41.4) | -15.4 (-30.5, -0.3) | 0.67 (0.48, 0.98) | 10.6 (9.7, 11.5) | -4.8 (-6.7, -2.9) | 0.69 (0.60, 0.79) |

*Per 10 000 person-years (95% confidence interval). AF: atrial fibrillation.

| **Table S10.** Association between decile of self-reported weekly MVPA as a categorical variable and all-cause dementia in general population | | | | | |
| --- | --- | --- | --- | --- | --- |
| **MVPA decile** | **MVPA**  **min/week** | **Hazard ratio**  **(95% CI)** | **P value** | **Hazard ratio**  **(95% CI)** | **P value** |
| 1 | 0 | Ref. | Ref. | 1.17 (1.04, 1.32) | 0.011 |
| 2 | 1-30 | 0.86 (0.74, 0.97) | 0.017 | 1.00 (0.86, 1.16) | 0.916 |
| 3 | 31-75 | 0.92 (0.82, 1.04) | 0.169 | 1.08 (0.95, 1.23) | 0.263 |
| 4 | 76-120 | 0.93 (0.83, 1.04) | 0.205 | 1.08 (0.95, 1.24) | 0.220 |
| 5 | 121-180 | 0.86 (0.76, 0.96) | 0.011 | Ref. | Ref. |
| 6 | 181-240 | 0.97 (0.86, 1.10) | 0.648 | 1.14 (0.99, 1.30) | 0.068 |
| 7 | 241-360 | 0.92 (0.82, 1.03) | 0.130 | 1.07 (0.94, 1.22) | 0.296 |
| 8 | 361-510 | 1.01 (0.89, 1.14) | 0.901 | 1.18 (1.03, 1.35) | 0.017 |
| 9 | 511-860 | 1.11 (0.99, 1.24) | 0.070 | 1.30 (1.14, 1.47) | < 0.001 |
| 10 | >861 | 1.18 (1.06, 1.31) | 0.003 | 1.38 (1.22, 1.56) | < 0.001 |

Cox regression was based on the fully model. MVPA: moderate-to-vigorous physical activity; CI: confidential interval.

| **Table S11.** Association between decile of self-reported weekly MVPA as a categorical variable and all-cause dementia in participants with atrial fibrillation | | | | | |
| --- | --- | --- | --- | --- | --- |
| **MVPA decile** | **MVPA**  **min/week** | **Hazard ratio**  **(95% CI)** | **P value** | **Hazard ratio**  **(95% CI)** | **P value** |
| 1 | 0 | Ref. | Ref. | 1.33 (0.79, 2.24) | 0.282 |
| 2 | 1-30 | 0.75 (0.41, 1.36) | 0.339 | 1.00 (0.50, 1.97) | 0.990 |
| 3 | 31-75 | 0.96 (0.61, 1.52) | 0.859 | 1.28 (0.72, 2.26) | 0.400 |
| 4 | 76-120 | 0.61 (0.36, 1.04) | 0.070 | 0.82 (0.43, 1.53) | 0.524 |
| 5 | 121-180 | 0.75 (0.45, 1.26) | 0.282 | Ref. | Ref. |
| 6 | 181-240 | 0.94 (0.56, 1.57) | 0.816 | 1.25 (0.68, 2.32) | 0.474 |
| 7 | 241-360 | 0.68 (0.40, 1.14) | 0.146 | 0.90 (0.49, 1.68) | 0.748 |
| 8 | 361-510 | 1.21 (0.77, 1.92) | 0.411 | 1.62 (0.91, 2.86) | 0.100 |
| 9 | 511-860 | 0.87 (0.53, 1.43) | 0.572 | 1.15 (0.63, 2.10) | 0.642 |
| 10 | >861 | 1.51 (0.99, 2.30) | 0.056 | 2.01 (1.17, 3.44) | 0.011 |

Cox regression was based on the fully model. MVPA: moderate-to-vigorous physical activity; CI: confidential interval.

| **Table S12.** Association between decile of self-reported weekly MVPA as a categorical variable and all-cause dementia in participants without atrial fibrillation | | | | | |
| --- | --- | --- | --- | --- | --- |
| **MVPA decile** | **MVPA**  **min/week** | **Hazard ratio**  **(95% CI)** | **P value** | **Hazard ratio**  **(95% CI)** | **P value** |
| 1 | 0 | Ref. | Ref. | 1.16 (1.03, 1.32) | 0.015 |
| 2 | 1-30 | 0.85 (0.74, 0.98) | 0.025 | 1.00 (0.86, 1.16) | 0.958 |
| 3 | 31-75 | 0.91 (0.81, 1.03) | 0.137 | 1.06 (0.93, 1.22) | 0.364 |
| 4 | 76-120 | 0.94 (0.84, 1.06) | 0.339 | 1.10 (0.96, 1.26) | 0.159 |
| 5 | 121-180 | 0.86 (0.76, 0.97) | 0.015 | Ref. | Ref. |
| 6 | 181-240 | 0.98 (0.86, 1.11) | 0.708 | 1.14 (0.99, 1.31) | 0.071 |
| 7 | 241-360 | 0.93 (0.83, 1.04) | 0.207 | 1.08 (0.95, 1.23) | 0.242 |
| 8 | 361-510 | 0.99 (0.88, 1.12) | 0.903 | 1.16 (1.01, 1.33) | 0.040 |
| 9 | 511-860 | 1.12 (1.00, 1.26) | 0.050 | 1.31 (1.15, 1.49) | < 0.001 |
| 10 | >861 | 1.15 (1.03, 1.29) | 0.011 | 1.35 (1.19, 1.53) | < 0.001 |

Cox regression was based on the fully model. MVPA: moderate-to-vigorous physical activity; CI: confidential interval.

| **Table S13.** Cox regression of physical activity and dementia risk grouped by sex in different population | | | | | | |
| --- | --- | --- | --- | --- | --- | --- |
|  | **Male** | | | **Female** | | |
|  | Event rate | HR (95% CI) | P value | Event rate | HR (95% CI) | P value |
| **All participants** | | | | | | |
| Below guideline | 1.79% | Ref. | Ref. | 1.00% | Ref. | Ref. |
| At/above guideline | 1.04% | 0.76 (0.62, 0.92) | 0.005 | 0.67% | 0.87 (0.71, 1.07) | 0.184 |
| **With AF** | | | | | | |
| Below guideline | 4.23% | Ref. | Ref. | 2.33% | Ref. | Ref. |
| At/above guideline | 2.38% | 0.63 (0.36, 1.09) | 0.101 | 1.97% | 0.69 (0.25, 1.86) | 0.463 |
| **Without AF** | | | | | | |
| Below guideline | 1.62% | Ref. | Ref. | 0.97% | Ref. | Ref. |
| At/above guideline | 0.98% | 0.77 (0.63, 0.95) | 0.016 | 0.66% | 0.88 (0.71, 1.08) | 0.219 |

Cox regression was based on the fully model. AF: atrial fibrillation; HR: hazard ratio; CI: confidential interval.

| **Table S14.** Associations between self-reported adherence of physical activity recommendations and all-cause dementia | | | | |
| --- | --- | --- | --- | --- |
|  | **Event rate** | **HR (95% CI)** | | |
|  |  | **Unadjusted model** | **Adjusted model** | **Compete model** |
| **All participants** | | | | |
| Below guideline | 1.62% | Ref. | Ref. | Ref. |
| At/above guideline | 1.65% | 1.05 (1.00, 1.11) | 1.07 (1.01, 1.14) | 1.04 (0.98, 1.10) |
| **With AF** | | | | |
| Below guideline | 5.26% | Ref. | Ref. | Ref. |
| At/above guideline | 4.93% | 0.97 (0.78, 1.21) | 1.09 (0.85, 1.39) | 1.07 (0.83, 1.38) |
| **Without AF** | | | | |
| Below guideline | 1.55% | Ref. | Ref. | Ref. |
| At/above guideline | 1.59% | 1.06 (1.01, 1.12) | 1.07 (1.01, 1.14) | 1.04 (0.97, 1.10) |

Cox regression was based on the fully model. AF: atrial fibrillation.

| **Table S15.** Association between decile of self-reported weekly MVPA as a categorical variable and all-cause dementia in general population | | | | | |
| --- | --- | --- | --- | --- | --- |
| **MVPA decile** | **MVPA**  **min/week** | **Hazard ratio**  **(95% CI)** | **P value** | **Hazard ratio**  **(95% CI)** | **P value** |
| 1 | 0 | Ref. | Ref. | 1.17 (1.04, 1.32) | 0.011 |
| 2 | 1-30 | 0.86 (0.74, 0.97) | 0.017 | 1.00 (0.86, 1.16) | 0.916 |
| 3 | 31-75 | 0.92 (0.82, 1.04) | 0.169 | 1.08 (0.95, 1.23) | 0.263 |
| 4 | 76-120 | 0.93 (0.83, 1.04) | 0.205 | 1.08 (0.95, 1.24) | 0.220 |
| 5 | 121-180 | 0.86 (0.76, 0.96) | 0.011 | Ref. | Ref. |
| 6 | 181-240 | 0.97 (0.86, 1.10) | 0.648 | 1.14 (0.99, 1.30) | 0.068 |
| 7 | 241-360 | 0.92 (0.82, 1.03) | 0.130 | 1.07 (0.94, 1.22) | 0.296 |
| 8 | 361-510 | 1.01 (0.89, 1.14) | 0.901 | 1.18 (1.03, 1.35) | 0.017 |
| 9 | 511-860 | 1.11 (0.99, 1.24) | 0.070 | 1.30 (1.14, 1.47) | < 0.001 |
| 10 | >861 | 1.18 (1.06, 1.31) | 0.003 | 1.38 (1.22, 1.56) | < 0.001 |

Cox regression was based on the fully model. MVPA: moderate-to-vigorous physical activity; CI: confidential interval.

| **Table S16.** Association between decile of self-reported weekly MVPA as a categorical variable and all-cause dementia in participants with atrial fibrillation | | | | | |
| --- | --- | --- | --- | --- | --- |
| **MVPA decile** | **MVPA**  **min/week** | **Hazard ratio**  **(95% CI)** | **P value** | **Hazard ratio**  **(95% CI)** | **P value** |
| 1 | 0 | Ref. | Ref. | 1.33 (0.79, 2.24) | 0.282 |
| 2 | 1-30 | 0.75 (0.41, 1.36) | 0.339 | 1.00 (0.50, 1.97) | 0.990 |
| 3 | 31-75 | 0.96 (0.61, 1.52) | 0.859 | 1.28 (0.72, 2.26) | 0.400 |
| 4 | 76-120 | 0.61 (0.36, 1.04) | 0.070 | 0.82 (0.43, 1.53) | 0.524 |
| 5 | 121-180 | 0.75 (0.45, 1.26) | 0.282 | Ref. | Ref. |
| 6 | 181-240 | 0.94 (0.56, 1.57) | 0.816 | 1.25 (0.68, 2.32) | 0.474 |
| 7 | 241-360 | 0.68 (0.40, 1.14) | 0.146 | 0.90 (0.49, 1.68) | 0.748 |
| 8 | 361-510 | 1.21 (0.77, 1.92) | 0.411 | 1.62 (0.91, 2.86) | 0.100 |
| 9 | 511-860 | 0.87 (0.53, 1.43) | 0.572 | 1.15 (0.63, 2.10) | 0.642 |
| 10 | >861 | 1.51 (0.99, 2.30) | 0.056 | 2.01 (1.17, 3.44) | 0.011 |

Cox regression was based on the fully model. MVPA: moderate-to-vigorous physical activity; CI: confidential interval.

| **Table S17.** Association between decile of self-reported weekly MVPA as a categorical variable and all-cause dementia in participants without atrial fibrillation | | | | | |
| --- | --- | --- | --- | --- | --- |
| **MVPA decile** | **MVPA**  **min/week** | **Hazard ratio**  **(95% CI)** | **P value** | **Hazard ratio**  **(95% CI)** | **P value** |
| 1 | 0 | Ref. | Ref. | 1.16 (1.03, 1.32) | 0.015 |
| 2 | 1-30 | 0.85 (0.74, 0.98) | 0.025 | 1.00 (0.86, 1.16) | 0.958 |
| 3 | 31-75 | 0.91 (0.81, 1.03) | 0.137 | 1.06 (0.93, 1.22) | 0.364 |
| 4 | 76-120 | 0.94 (0.84, 1.06) | 0.339 | 1.10 (0.96, 1.26) | 0.159 |
| 5 | 121-180 | 0.86 (0.76, 0.97) | 0.015 | Ref. | Ref. |
| 6 | 181-240 | 0.98 (0.86, 1.11) | 0.708 | 1.14 (0.99, 1.31) | 0.071 |
| 7 | 241-360 | 0.93 (0.83, 1.04) | 0.207 | 1.08 (0.95, 1.23) | 0.242 |
| 8 | 361-510 | 0.99 (0.88, 1.12) | 0.903 | 1.16 (1.01, 1.33) | 0.040 |
| 9 | 511-860 | 1.12 (1.00, 1.26) | 0.050 | 1.31 (1.15, 1.49) | < 0.001 |
| 10 | >861 | 1.15 (1.03, 1.29) | 0.011 | 1.35 (1.19, 1.53) | < 0.001 |

Cox regression was based on the fully model. MVPA: moderate-to-vigorous physical activity; CI: confidential interval.

| Table S18. Subgroup analysis of the adherence of physical activity recommendations and all-cause dementia | | | | | | | | | |
| --- | --- | --- | --- | --- | --- | --- | --- | --- | --- |
| Subgroup | **All participants** | | | **With atrial fibrillation** | | | **Without atrial fibrillation** | | |
|  | **HR (95% CI)** | **P value** | **P interaction** | **HR (95% CI)** | **P value** | **P interaction** | **HR (95% CI)** | **P value** | **P interaction** |
| Age | | | | | | | | | |
| < 60 years | 0.77 (0.54, 1.09) | 0.139 | 0.478 | 0.60 (0.36, 0.99) | 0.048 | 0.584 | 0.76 (0.53, 1.08) | 0.128 | 0.394 |
| ≥ 60 years | 0.82 (0.70, 0.96) | 0.012 |  | 0.59 (0.07, 4.89) | 0.625 |  | 0.84 (0.71, 0.99) | 0.044 |  |
| Sex | | | | | | | | | |
| Male | 0.76 (0.62, 0.92) | 0.007 | 0.424 | 0.63 (0.36, 1.09) | 0.101 | 0.745 | 0.77 (0.63, 0.95) | 0.016 | 0.515 |
| Female | 0.87 (0.71, 1.07) | 0.184 |  | 0.69 (0.25, 1.86) | 0.463 |  | 0.88 (0.71, 1.08) | 0.219 |  |
| Employment status | | | | | | | | | |
| Employed | 0.85 (0.72, 1.01) | 0.052 | 0.224 | 0.69 (0.40, 1.19) | 0.179 | 0.464 | 0.87 (0.73, 1.04) | 0.118 | 0.289 |
| Others | 0.69 (0.54, 0.90) | 0.006 |  | 0.59 (0.22, 1.59) | 0.295 |  | 0.71 (0.54, 0.92) | 0.011 |  |
| Body mass index | | | | | | | | | |
| < 28 | 0.83 (0.69, 0.98) | 0.030 | 0.591 | 0.58 (0.32, 1.06) | 0.075 | 0.774 | 0.85 (0.71, 1.02) | 0.083 | 0.521 |
| ≥ 28 | 0.75 (0.59, 0.97) | 0.028 |  | 0.88 (0.38, 2.08) | 0.780 |  | 0.75 (0.58, 0.97) | 0.030 |  |
| Hypertension | | | | | | | | | |
| Yes | 0.79 (0.64, 0.98) | 0.032 | 0.734 | 0.49 (0.26, 0.93) | 0.029 | 0.156 | 0.85 (0.68, 1.07) | 0.168 | 0.875 |
| No | 0.84 (0.69, 1.01) | 0.060 |  | 1.15 (0.50, 2.67) | 0.740 |  | 0.82 (0.68, 0.99) | 0.047 |  |
| Diabetes | | | | | | | | | |
| Yes | 0.61 (0.38, 0.98) | 0.042 | 0.134 | 0.55 (0.33, 0.91) | 0.021 | 0.168 | 0.50 (0.30, 0.84) | 0.008 | 0.039 |
| No | 0.83 (0.72, 0.96) | 0.015 |  | 1.38 (0.38, 4.98) | 0.622 |  | 0.87 (0.74, 1.01) | 0.070 |  |
| Cardiovascular diseases | | | | | | | | | |
| Yes | 0.81 (0.57, 1.14) | 0.223 | 0.761 | 0.71 (0.36, 1.41) | 0.328 | 0.909 | 0.86 (0.57, 1.29) | 0.468 | 0.603 |
| No | 0.81 (0.69, 0.94) | 0.006 |  | 0.61 (0.30, 1.23) | 0.165 |  | 0.82 (0.70, 0.96) | 0.012 |  |
| Stroke | | | | | | | | | |
| Yes | 0.78 (0.38, 1.63) | 0.509 | 0.768 | - | - | 0.917 | 0.73 (0.31, 1.72) | 0.465 | 0.650 |
| No | 0.81 (0.70, 0.93) | 0.004 |  | 0.65 (0.39, 1.09) | 0.103 |  | 0.83 (0.71, 0.96) | 0.012 |  |

Cox regression was based on the fully model. HR: hazard ratio; CI: confidential interval.

**
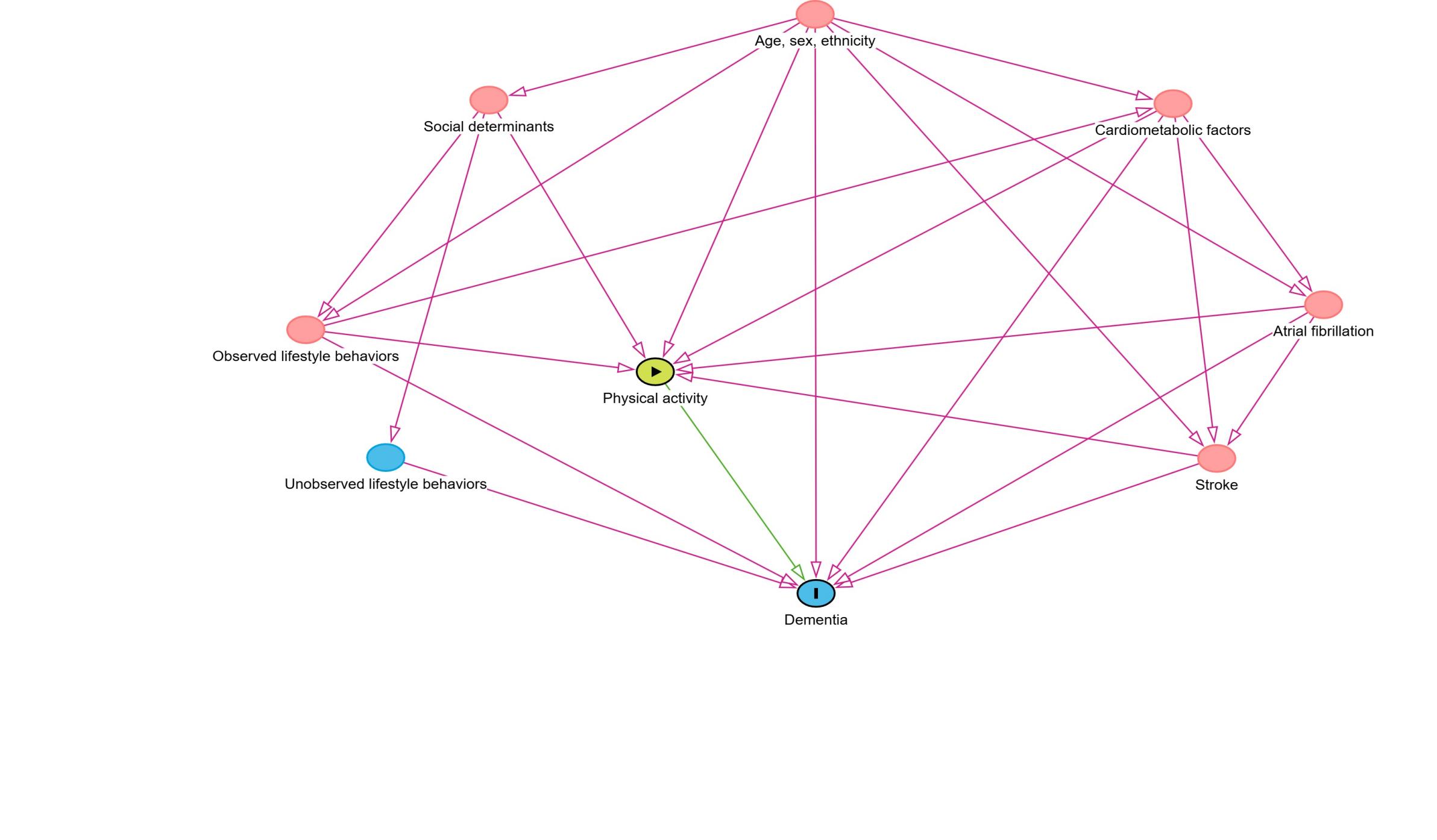
**

**Figure S1.** Directed Acyclic Graph displaying the relationship between physical activity, incident dementia and other variables. Social determinants: employment status, education score, health score, Townsend deprivation index; Observed lifestyle behaviors: smoking, drinking; Cardio-metabolic factors: body mass index, hypertension, diabetes, other cardiovascular diseases.


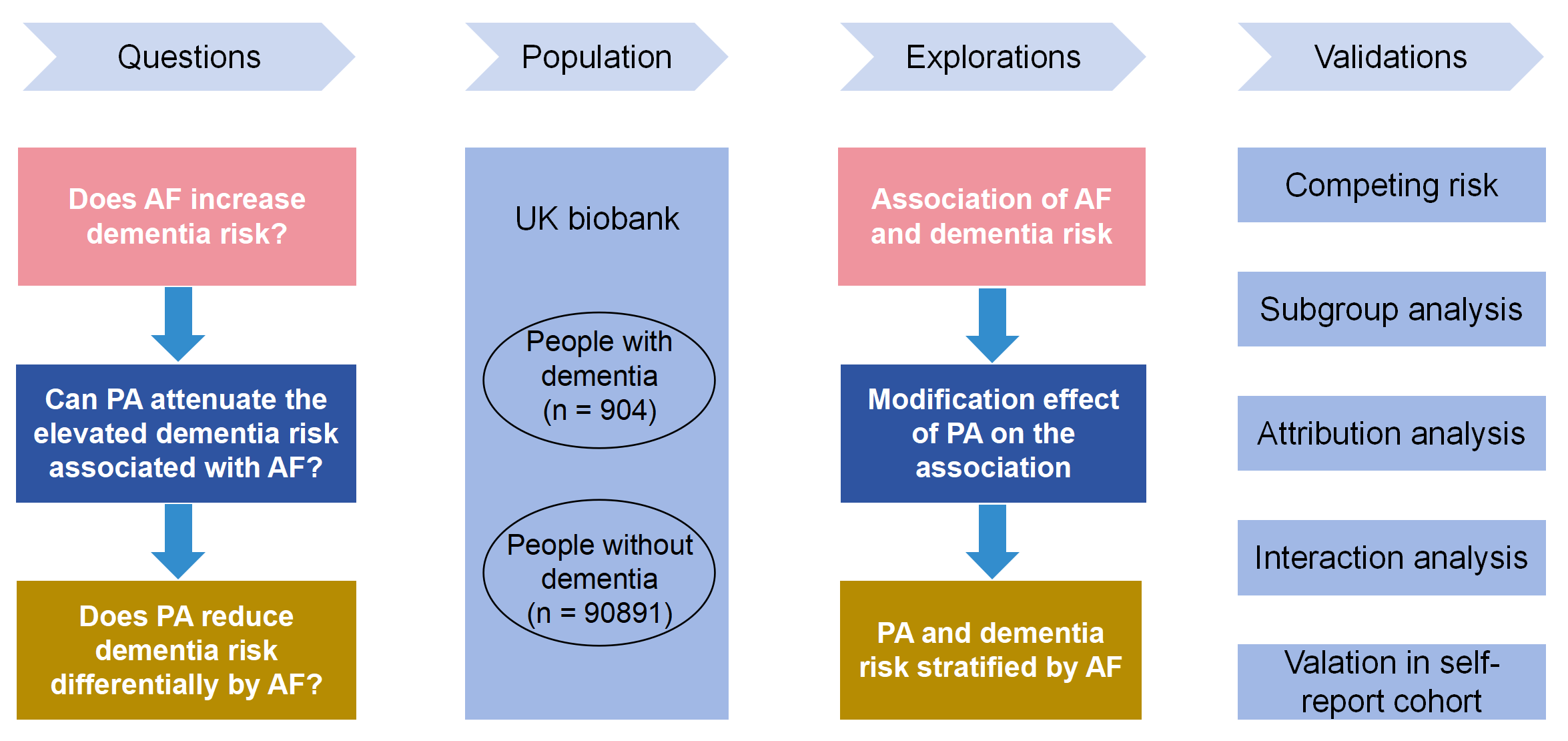


Figure S2. Design of this study. AF: atrial fibrillation; PA: physical activity.

**
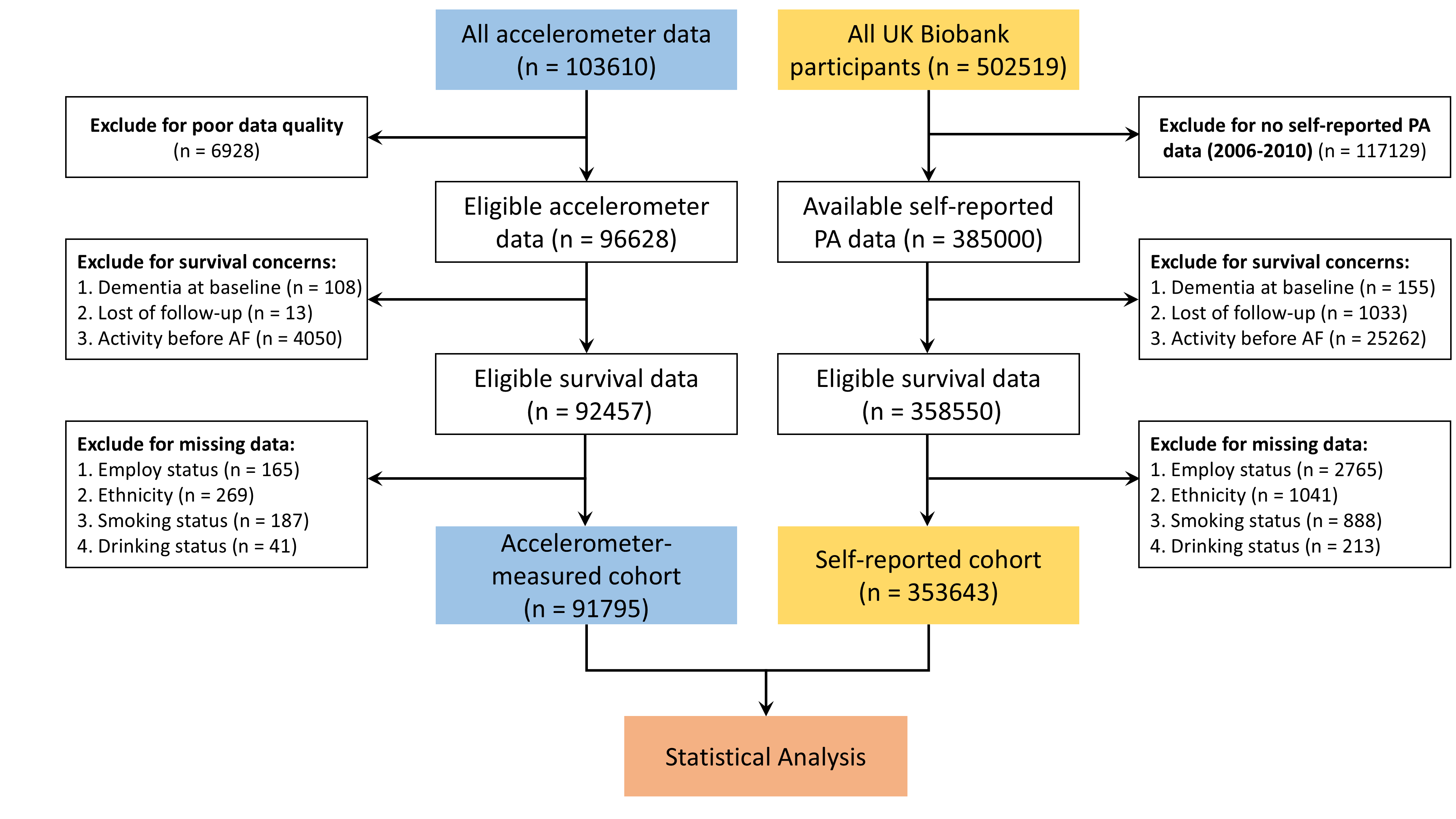
**

**Figure S3.** Flow chart. AF: atrial fibrillation

**
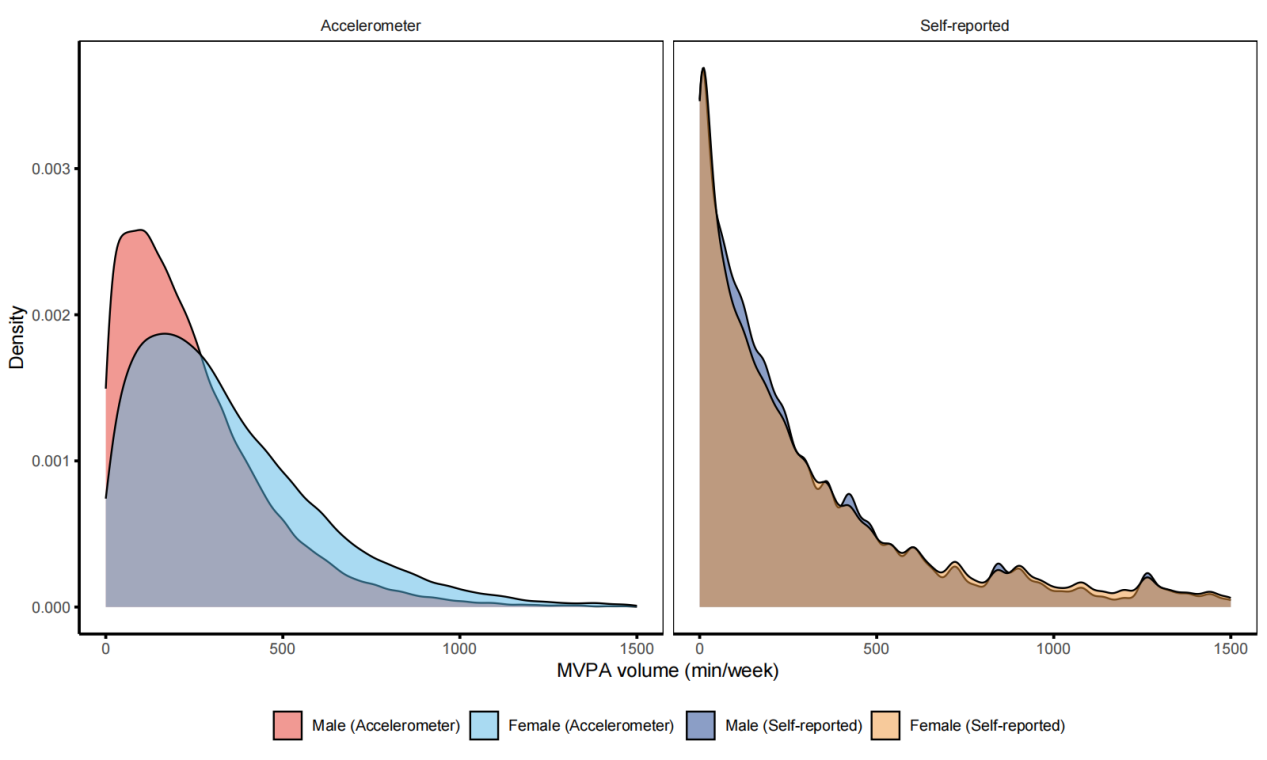
**

**Figure S4.** Distribution of physical activity volume.

**
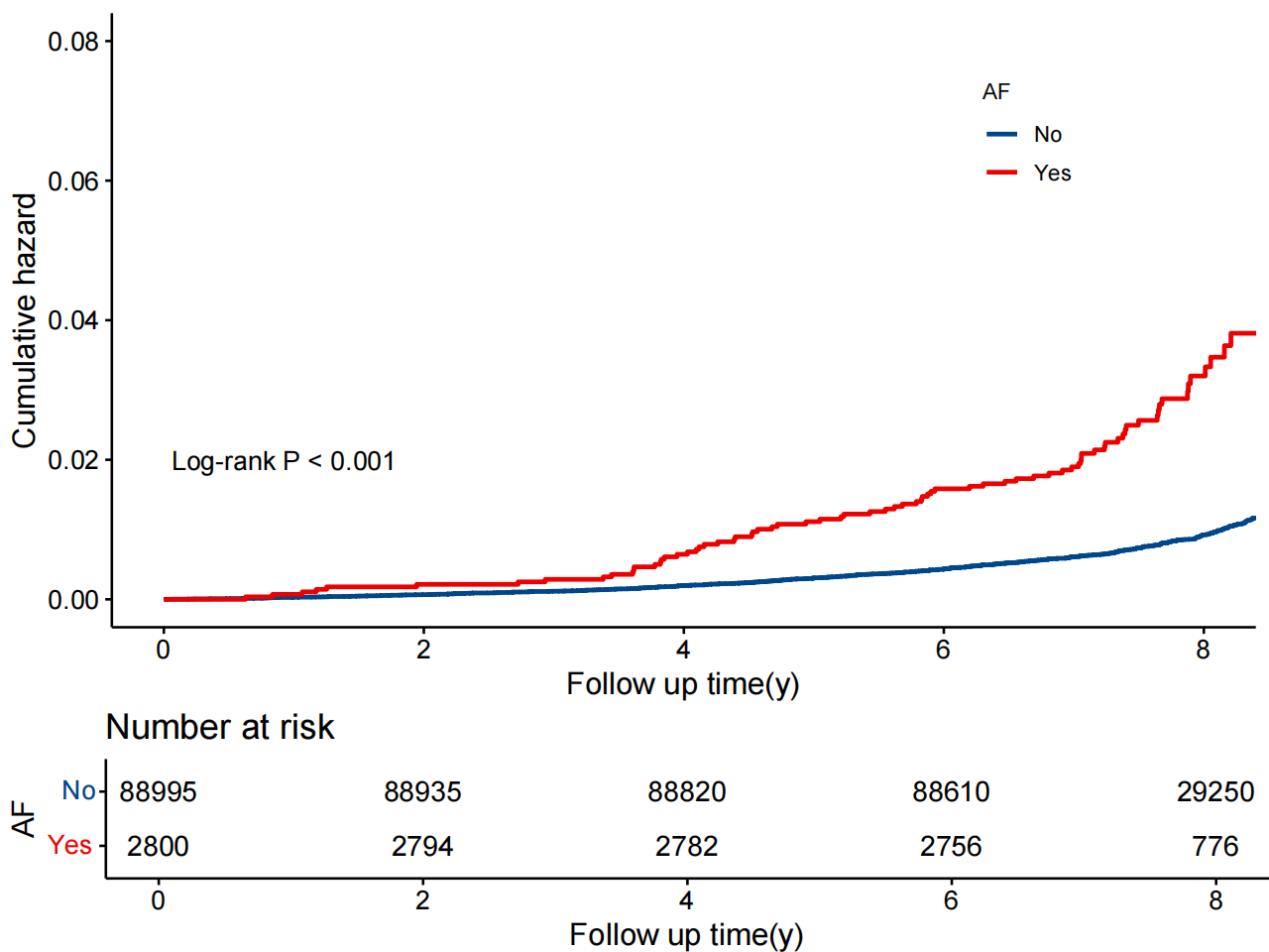
**

**Figure S5.** Cumulative risk of dementia among groups with and without atrial fibrillation (AF).

**
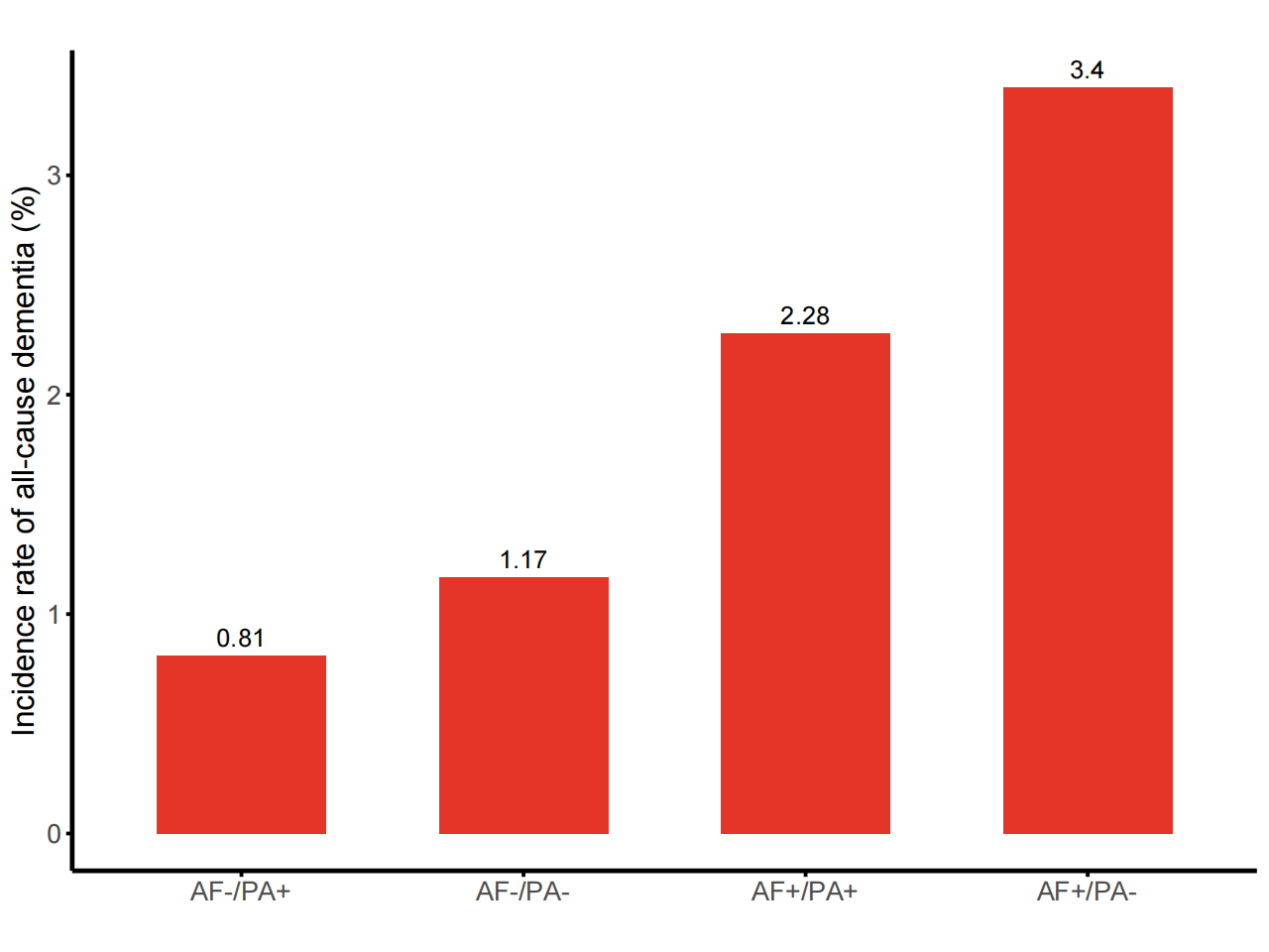
**

**Figure S6.** The incidence rate of all-cause dementia across different AF and physical activity status.

**
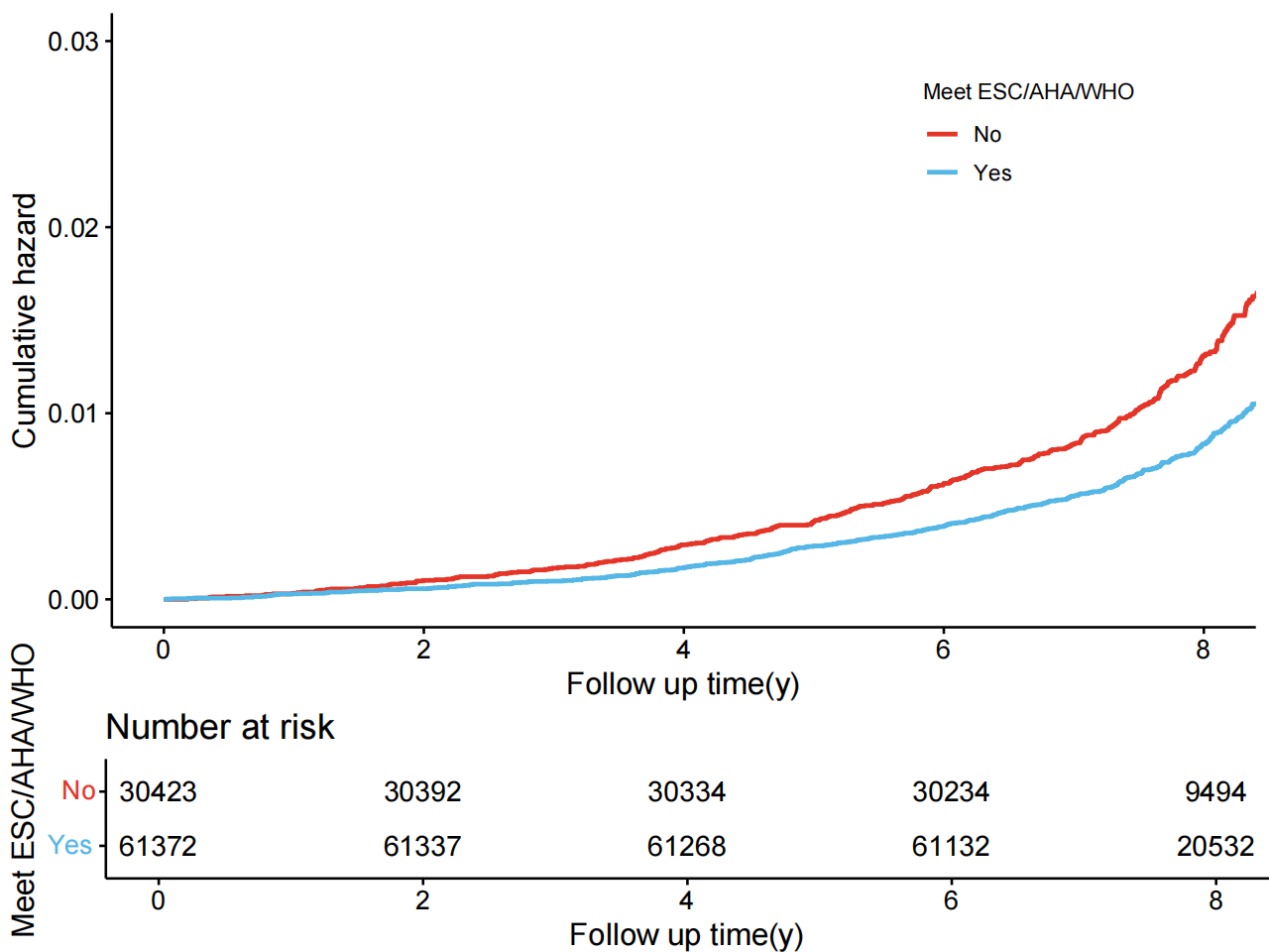
**

**Figure S7.** Cumulative risk of dementia among groups stratified by adherence to physical activity recommendations in the general population.


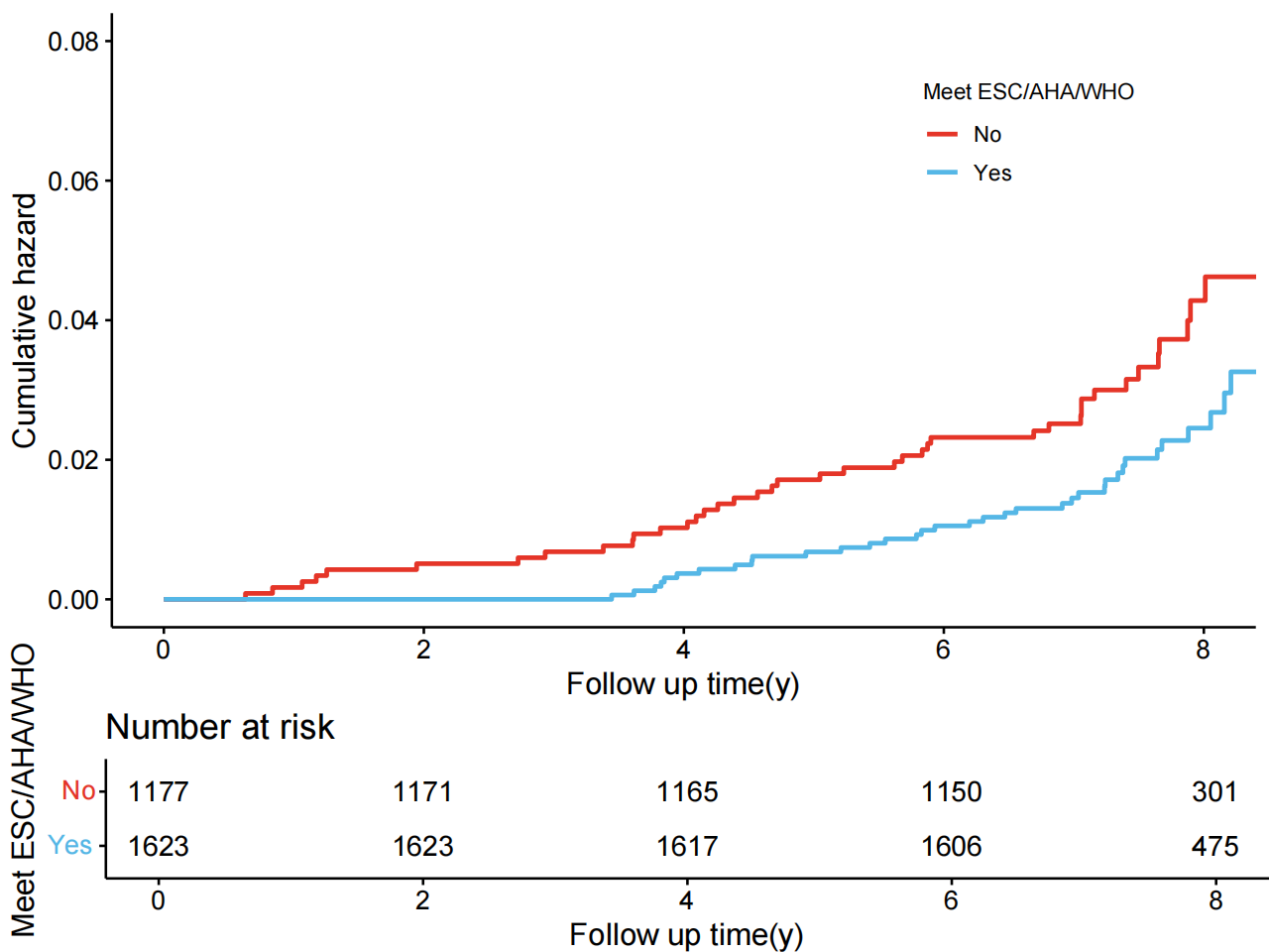
**Figure S8.** Cumulative risk of dementia among groups stratified by adherence to physical activity recommendations in patients with atrial fibrillation.


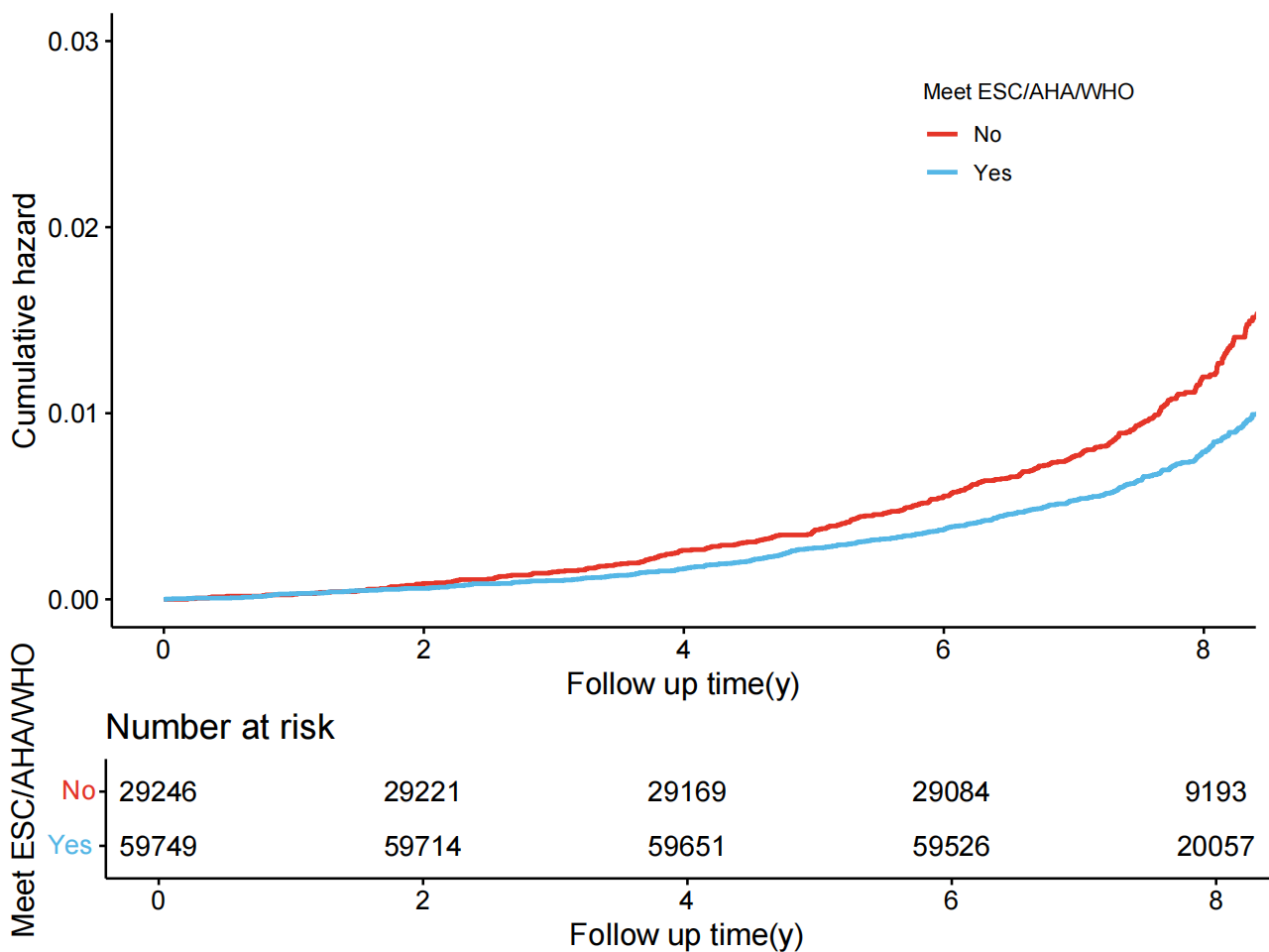
**Figure S9.** Cumulative risk of dementia among groups stratified by adherence to physical activity recommendations in patients without atrial fibrillation.


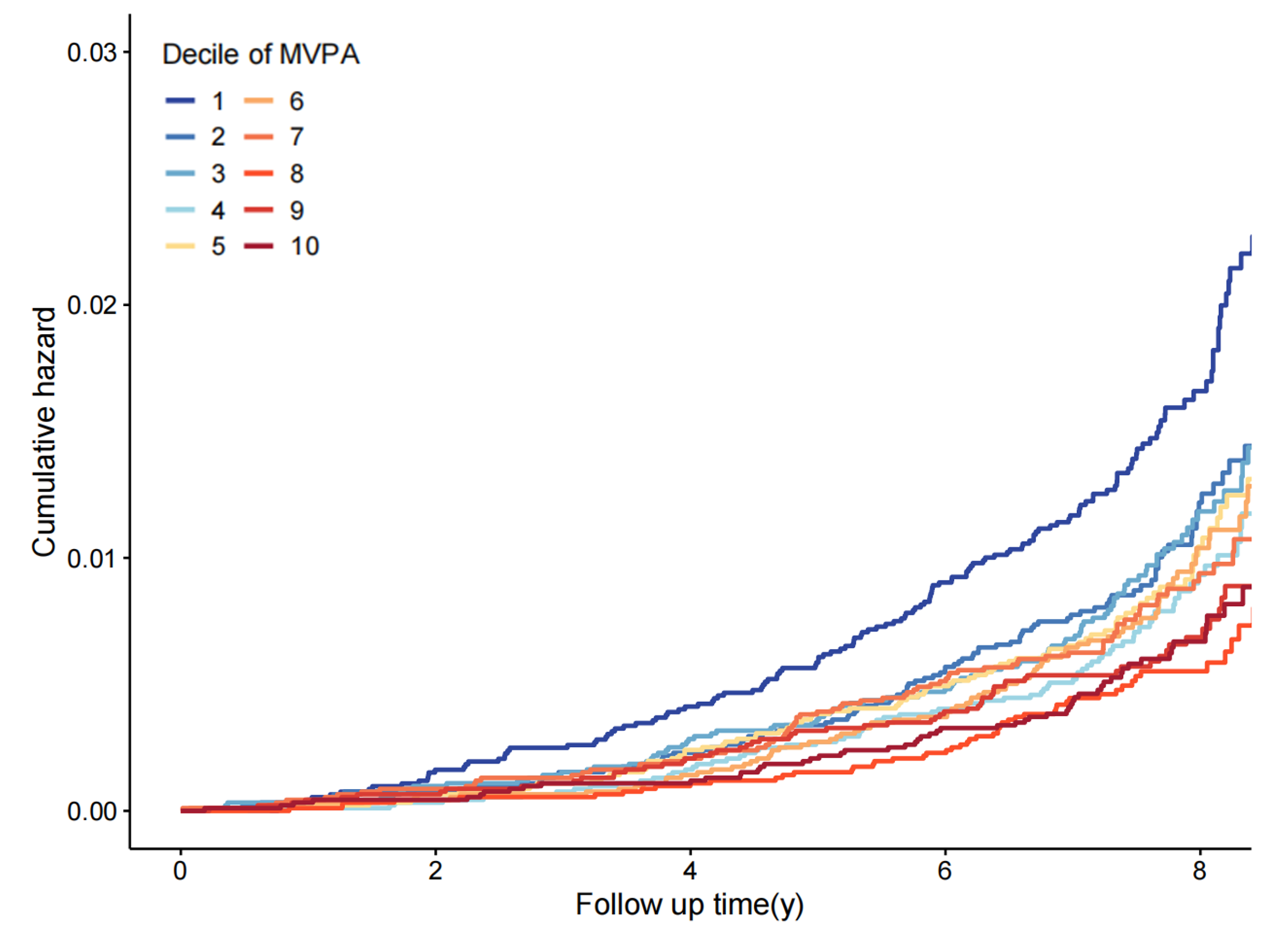


**Figure S10.** Cumulative risk of all-cause dementia stratified by decile of accelerometer measured moderate-to-vigorous physical activity.


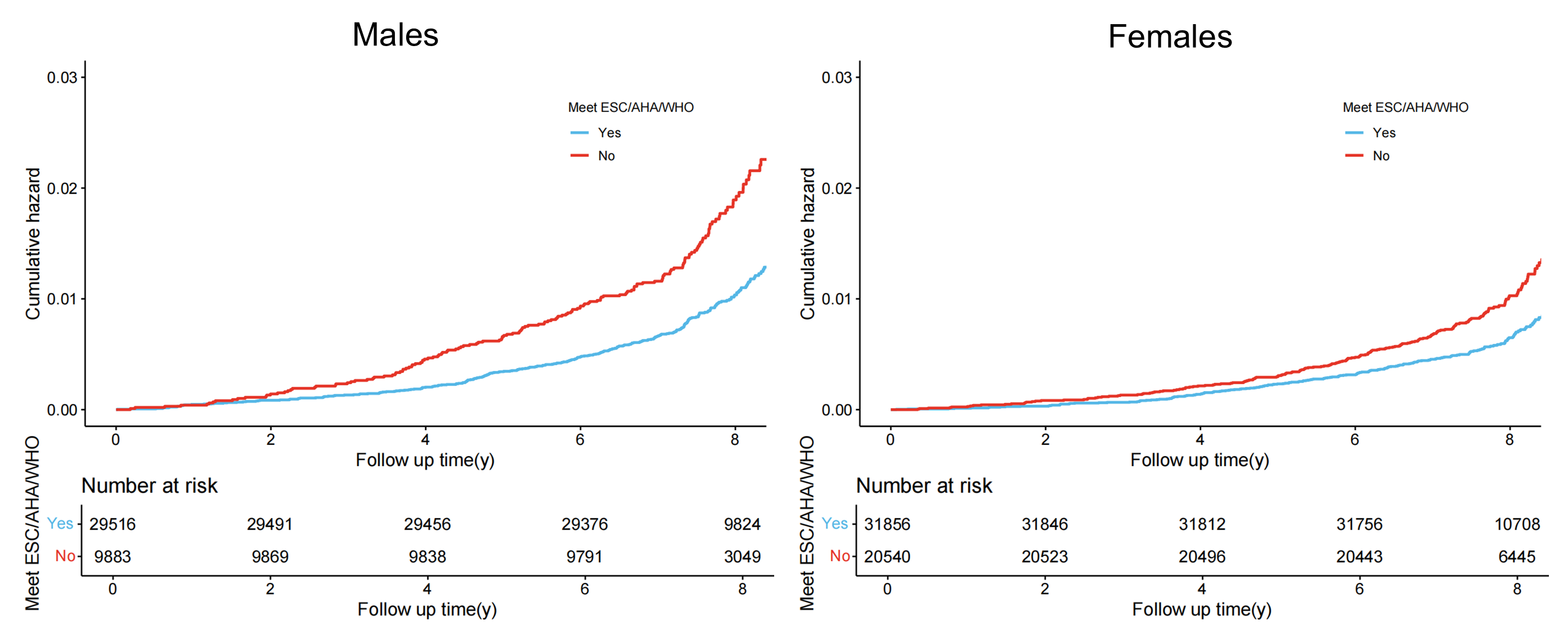


**Figure S11.** Cumulative risk of dementia among groups stratified by adherence to physical activity recommendations in the general population for males (left) and females (right).


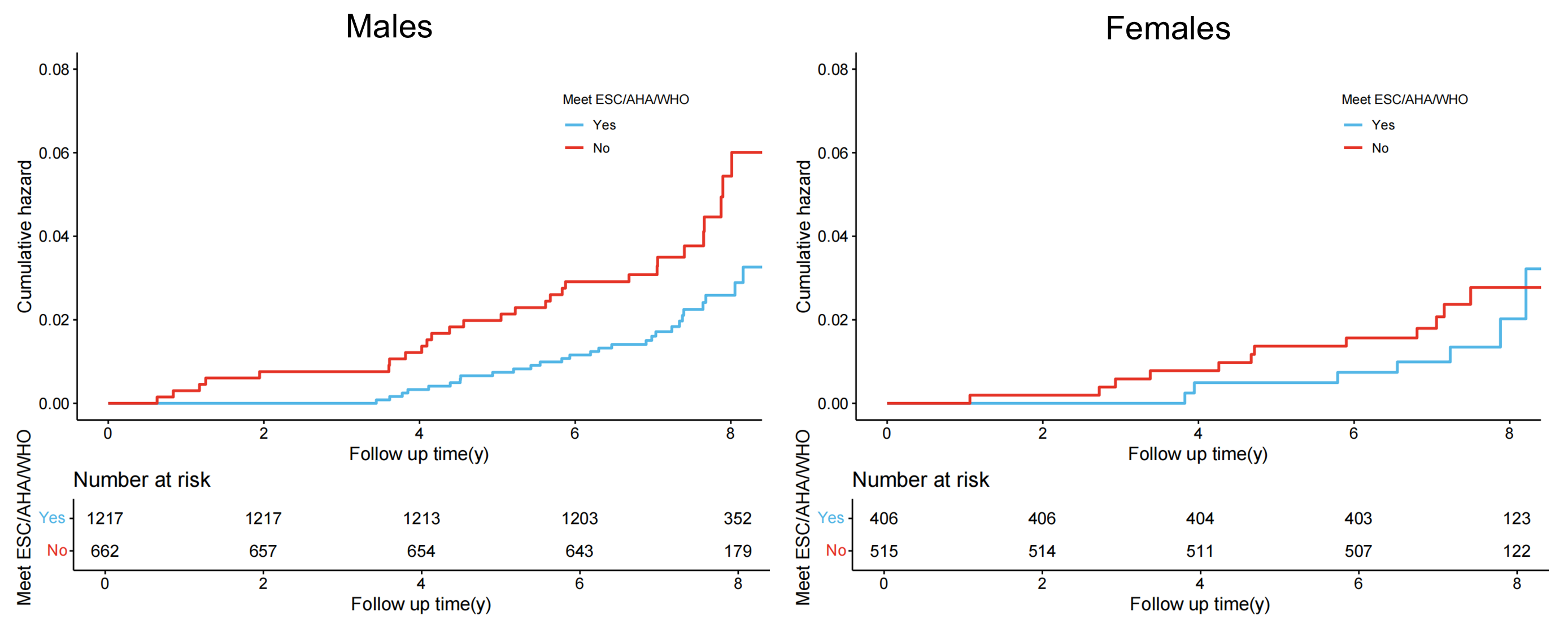


**Figure S12.** Cumulative risk of dementia among groups stratified by adherence to physical activity recommendations in participants with atrial fibrillation for males (left) and females (right).


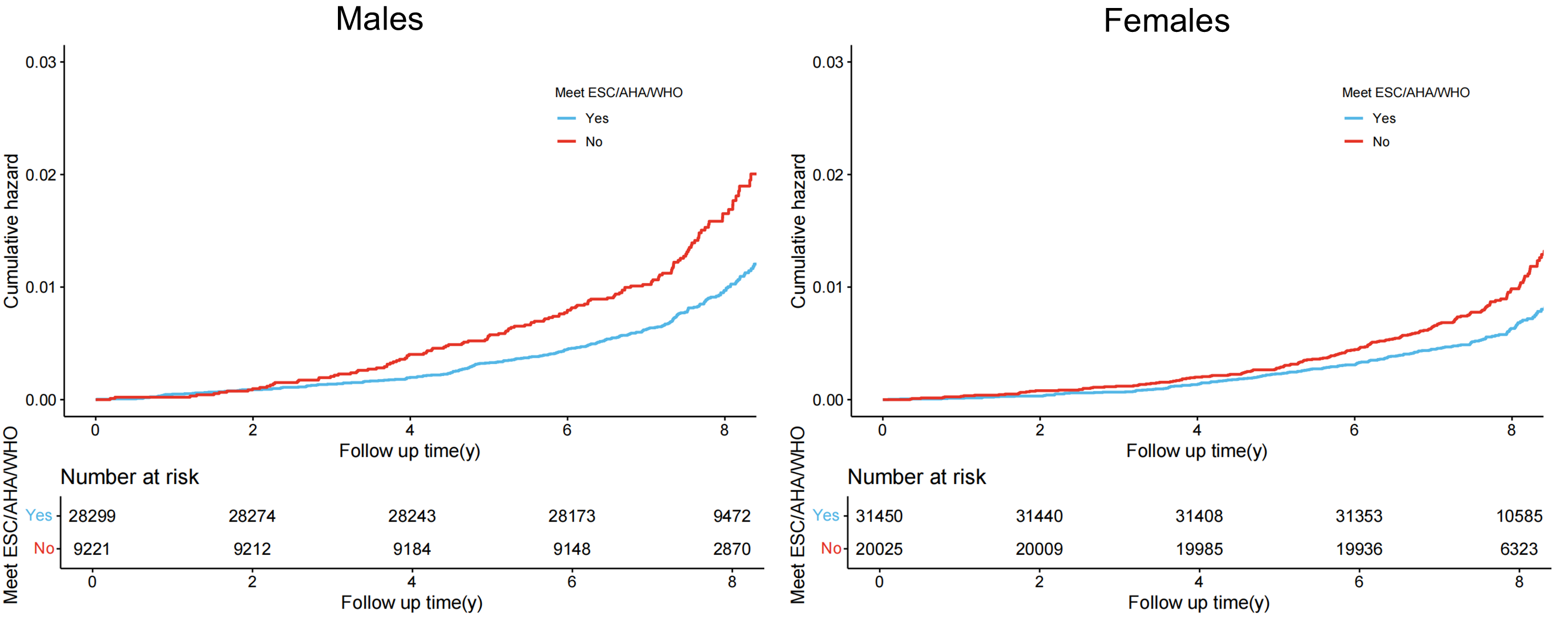


**Figure S13.** Cumulative risk of dementia among groups stratified by adherence to physical activity recommendations in participants without atrial fibrillation for males (left) and females (right).


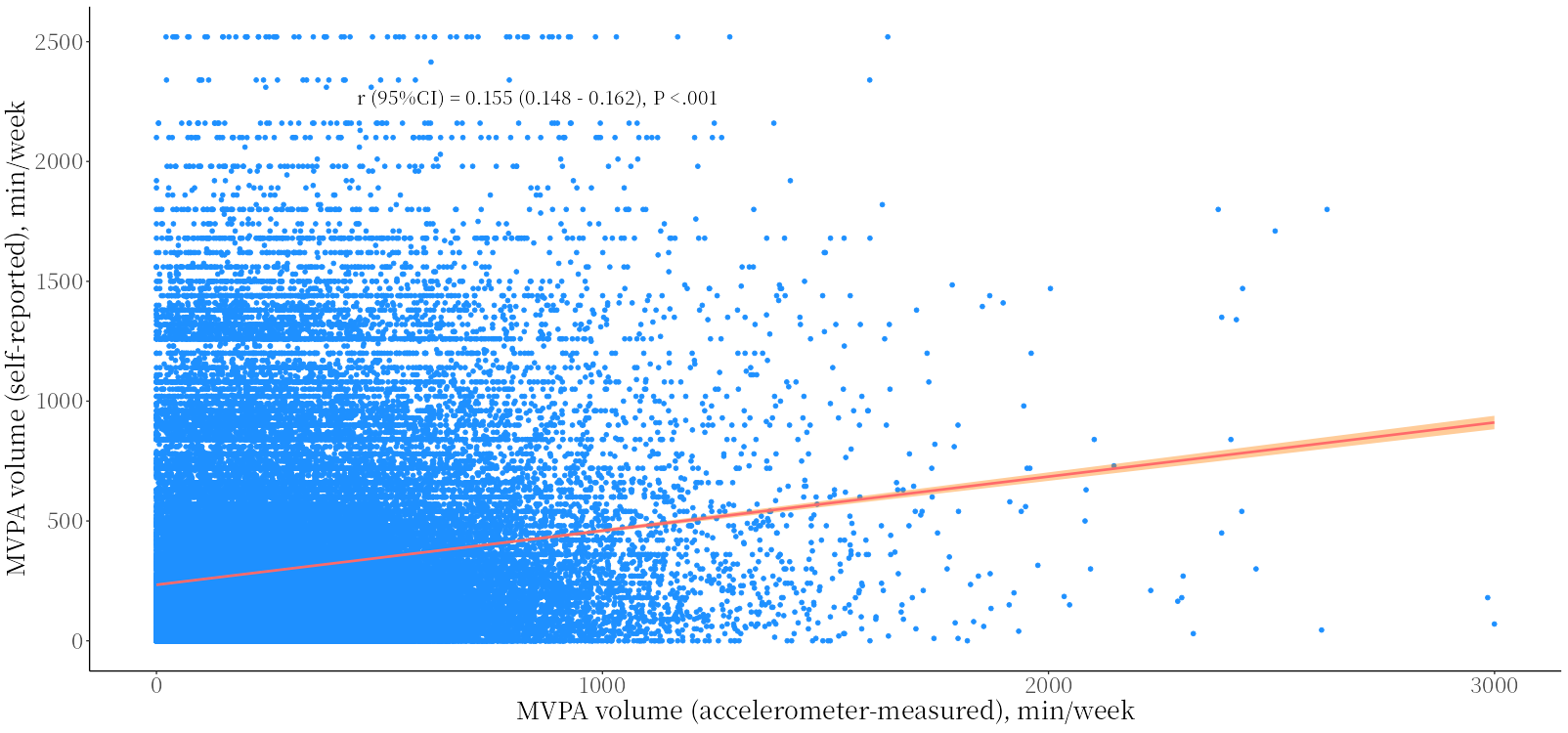


**Figure S14.** Correlation of accelerometer-measured and self-reported physical activity volume. MVPA: moderate-to-vigorous physical activity.

**
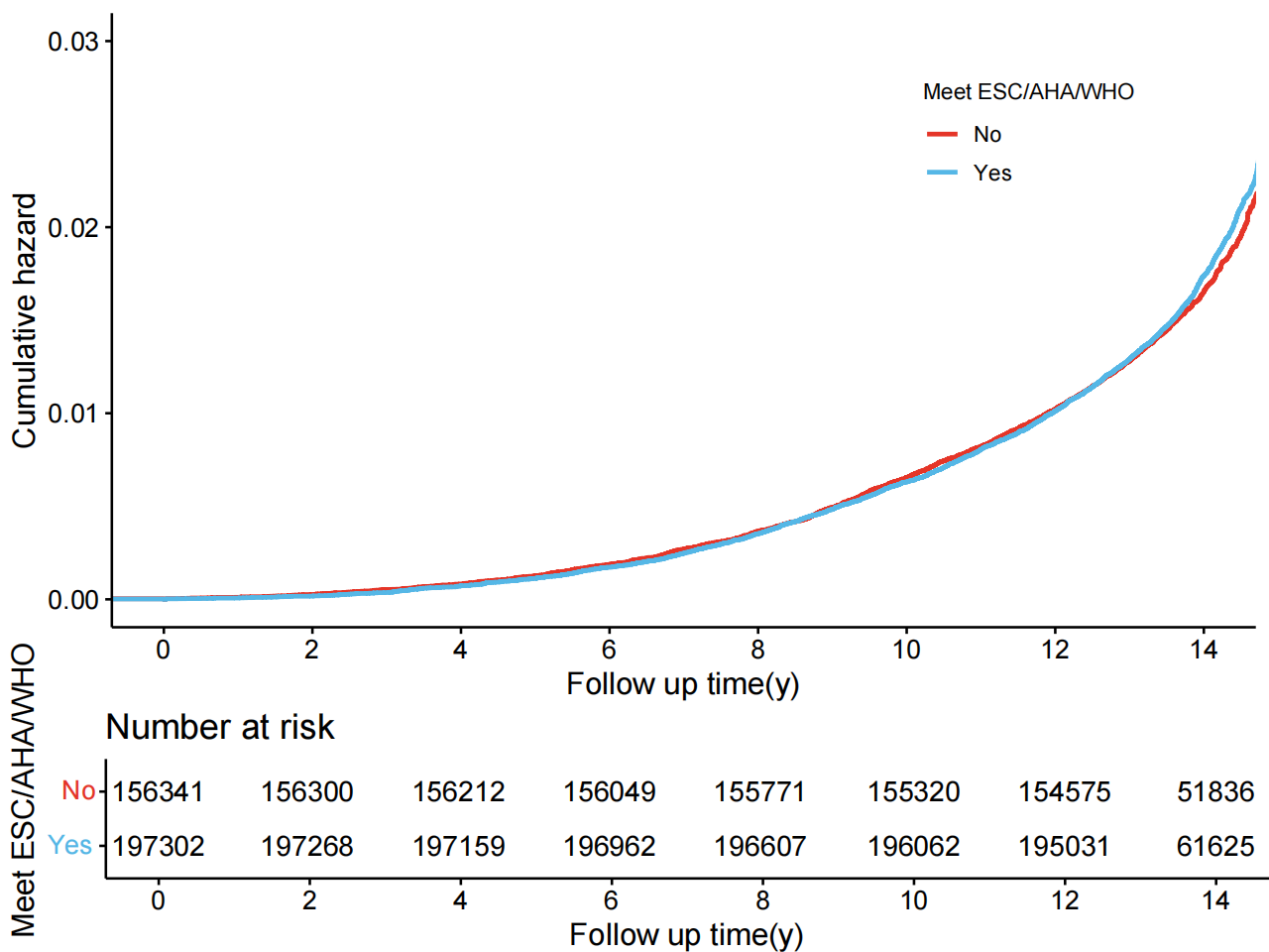
Figure S15.** Cumulative risk of dementia among self-reported groups stratified by adherence to physical activity recommendations.

**
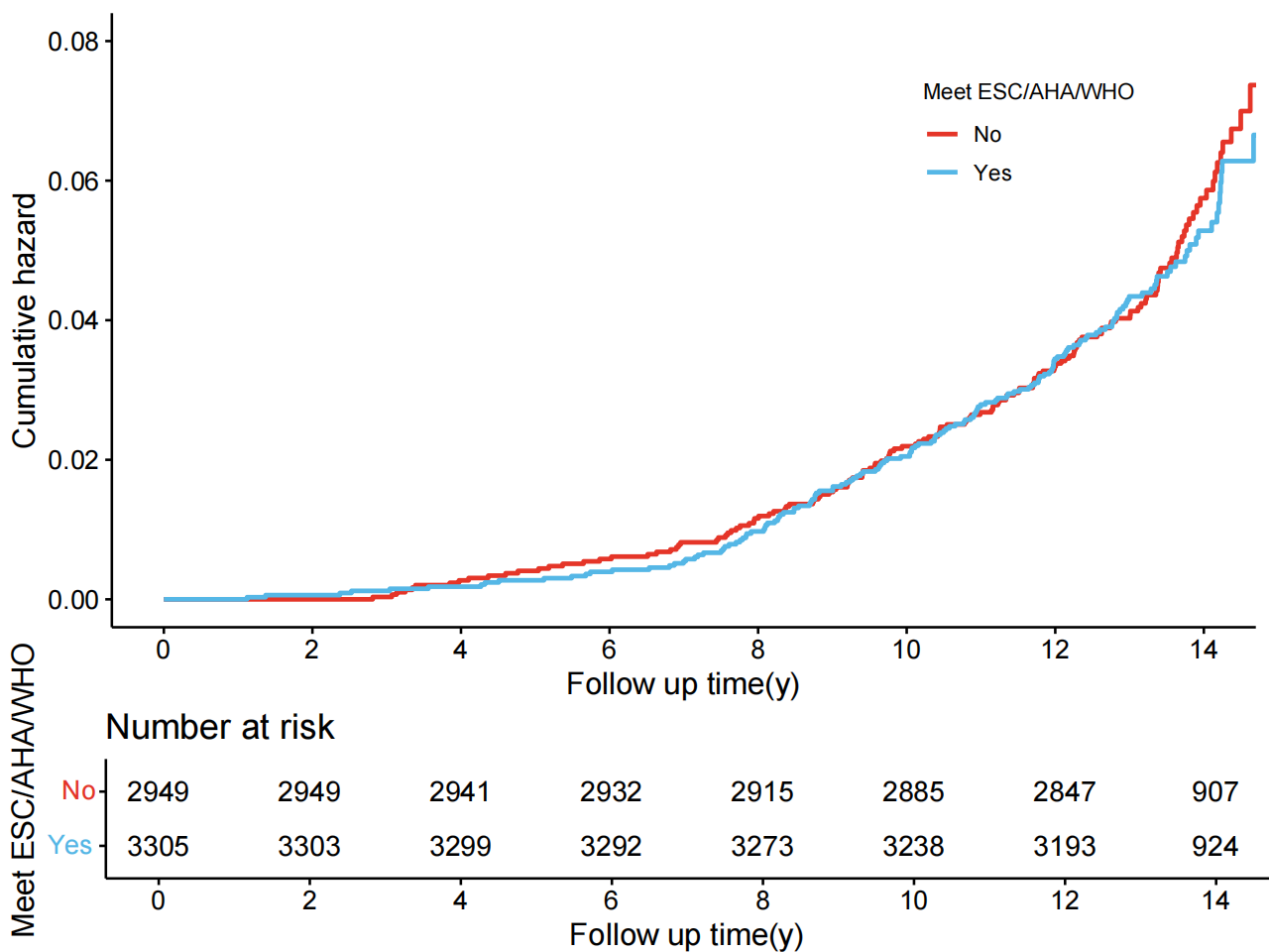
Figure S16.** Cumulative risk of dementia among self-reported groups stratified by adherence to physical activity recommendations in participants with atrial fibrillation.

**
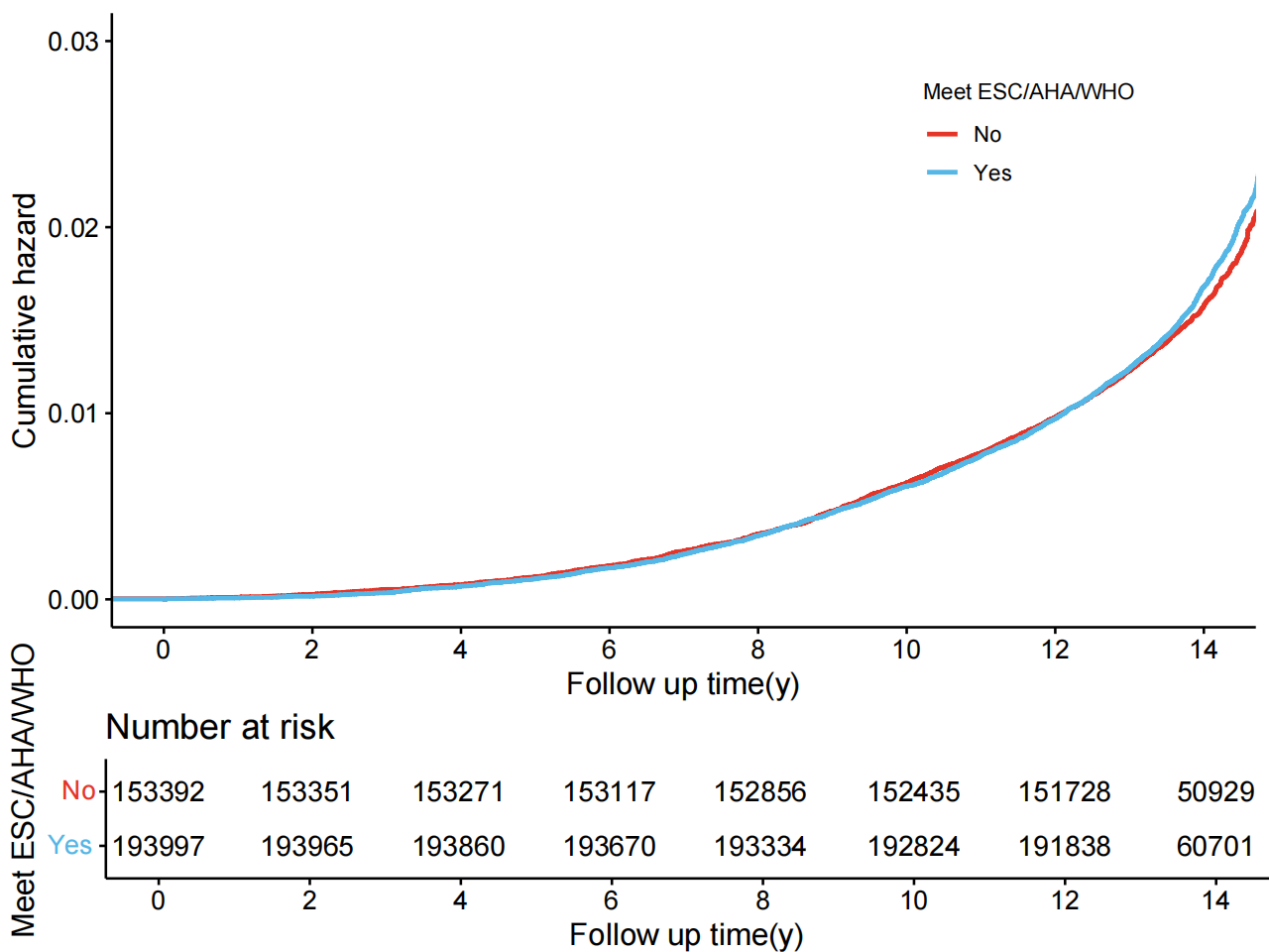
Figure S17.** Cumulative risk of dementia among self-reported groups stratified by adherence to physical activity recommendations in participants without atrial fibrillation.


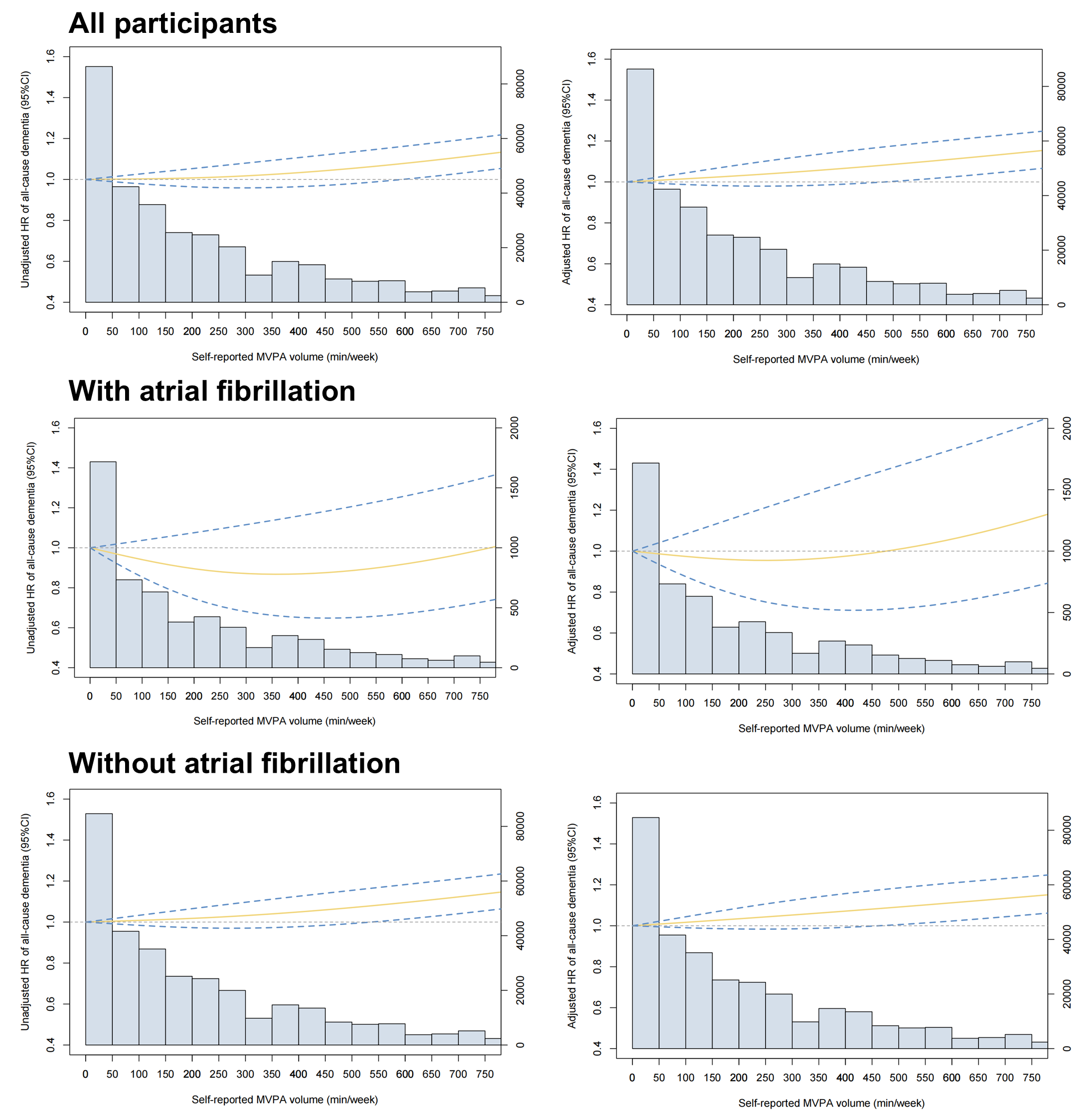


**Figure S18.** Dose-response relationship between self-reported moderate to vigorous physical activity volume and risk of all-cause dementia in different population.

**
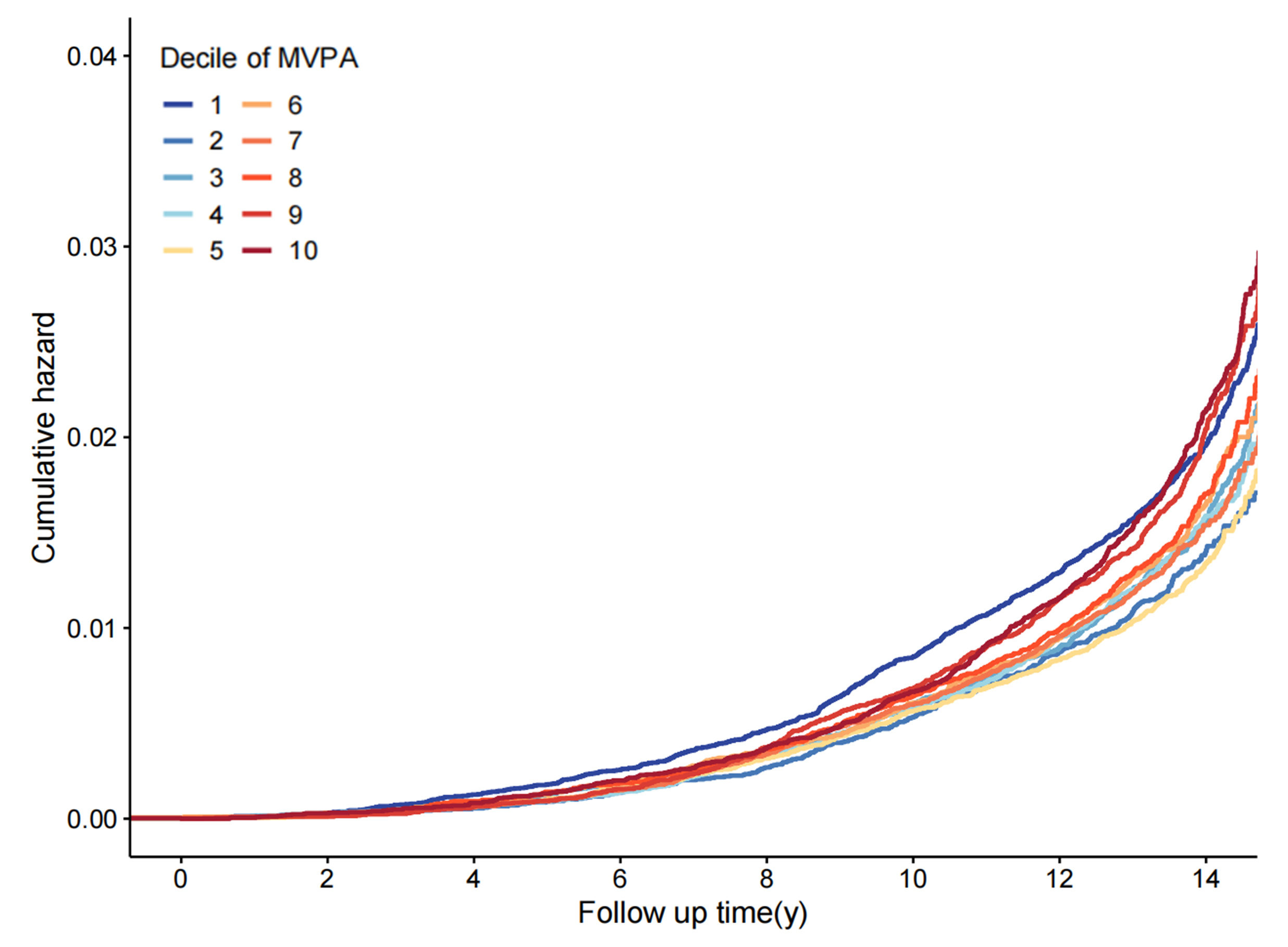
Figure S19.** Cumulative risk of all-cause dementia stratified by decile of self-reported moderate-to-vigorous physical activity.
